# A critical regulator of Bcl2 revealed by systematic transcript discovery of lncRNAs associated with T-cell differentiation

**DOI:** 10.1038/s41598-019-41247-5

**Published:** 2019-03-18

**Authors:** Wiam Saadi, Yasmina Kermezli, Lan T. M. Dao, Evelyne Mathieu, David Santiago-Algarra, Iris Manosalva, Magali Torres, Mohamed Belhocine, Lydie Pradel, Beatrice Loriod, Mourad Aribi, Denis Puthier, Salvatore Spicuglia

**Affiliations:** 1 Aix-Marseille University, Inserm, TAGC, UMR1090 Marseille, France; 2 Equipe Labélisée Ligue Contre le Cancer, Marseille, France; 3 Laboratory of Applied Molecular Biology and Immunology, W0414100, University of Tlemcen, Tlemcen, Algeria; 4 Present Address: Vinmec Research Institute of Stem cell and Gene technology (VRISG), Hanoi, Vietnam; 5 Present Address: Molecular Biology and Genetics Laboratory, Dubai, United Arab Emirates

## Abstract

Normal T-cell differentiation requires a complex regulatory network which supports a series of maturation steps, including lineage commitment, T-cell receptor (TCR) gene rearrangement, and thymic positive and negative selection. However, the underlying molecular mechanisms are difficult to assess due to limited T-cell models. Here we explore the use of the pro-T-cell line P5424 to study early T-cell differentiation. Stimulation of P5424 cells by the calcium ionophore ionomycin together with PMA resulted in gene regulation of T-cell differentiation and activation markers, partially mimicking the CD4^-^CD8^-^ double negative (DN) to double positive (DP) transition and some aspects of subsequent T-cell maturation and activation. Global analysis of gene expression, along with kinetic experiments, revealed a significant association between the dynamic expression of coding genes and neighbor lncRNAs including many newly-discovered transcripts, thus suggesting potential co-regulation. CRISPR/Cas9-mediated genetic deletion of *Robnr*, an inducible lncRNA located downstream of the anti-apoptotic gene *Bcl2*, demonstrated a critical role of the *Robnr* locus in the induction of *Bcl2*. Thus, the pro-T-cell line P5424 is a powerful model system to characterize regulatory networks involved in early T-cell differentiation and maturation.

## Introduction

T lymphocytes are one of the main players of the adaptive immunity. T-cell development in the thymus requires temporally regulated rearrangements of the T-cell receptor (*Tcr*) genes and a series of selection events, whereby newly assembled TCR complexes signal for cell survival, proliferation and differentiation processes^1,2^. The *Tcrb* locus rearranges in the most immature thymocytes, known as CD4^−^CD8^−^ double-negative (DN) thymocytes. Thymocytes that have successfully rearranged a *Tcrb* allele differentiate into CD4^+^CD8^+^ double-positive (DP) thymocytes in a process known as β-selection. This process is driven by signaling through the pre-TCR, which is composed of TCRβ and the invariant pTα protein, and through cooperation with the Notch signaling pathway^1,3^. The β-selection process triggers the activation of *Tcra* rearrangements and transcription along with complex intracellular pathways resulting in wide changes in the transcriptional and epigenetic programs of the immature T cells^4–6^. The expression of a functionally rearranged *Tcra* gene leads to the formation of a variable TCRαβ heterodimer and, ultimately, to the selection of TCRαβ expressing cells which will terminally differentiate into CD4^+^ or CD8^+^ single positive (SP) T cells. Disruptions of these genetic and epigenetic processes might result in oncogenic transformation of T-cell precursors (*i*.*e*. leukemia and lymphoma^7,8^) or immune-related pathologies^9^.

Long non-coding RNAs (lncRNAs) are a heterogeneous group of non-coding genes transcribed by RNA polymerase II from intergenic or intragenic regions and varying in length from 200 nt to over 100 kb^10^. Many studies have demonstrated that lncRNAs are key components of the repertoire of regulatory elements that control normal development and disease^11–13^. Although the mechanism(s) of regulation of gene expression by lncRNAs are not yet well understood, many of them have been shown to mediate epigenetic modifications by recruiting chromatin remodeling complexes to specific loci, thus regulating both the expression of neighboring genes and distant genomic sequences^14^.

Several lncRNAs involved in normal and malignant hematopoiesis have been discovered^15–17^, including lncRNAs involved in T-cell differentiation and activation^18–22^, T-cell immune response^23–25^, lymphoid malignancies^26–32^ and immuno-deficiencies^23,33^. As the expression of lncRNAs is highly tissue- and context-specific, it is likely that many relevant lncRNAs involved in T-cell lymphocyte differentiation and function have not yet been identified.

To gain insights into the lncRNAs regulatory circuits that underlie T lymphocyte differentiation and activation we used the pro-T-cell line P5424 as a tractable model^34^. The P5424 cell line has been derived from *Rag1*^−/−^; *p53*^−/−^ mice and express the CD4 and CD8 markers, but also early T-cell markers, such as *Hes1* and *Ptcra*, which encodes for the surrogate α chain (pTα) of the pre-TCR complex, suggesting that the cells are blocked at the β-selection checkpoint^5,34,35^. Combined treatment of T cells with phorbol 12-myristate 13-acetate (PMA) and ionomycin have been shown to resemble (pre-)TCR mediated signaling by synergistic activation of calcium ions (Ca^+^) and mitogen-activated protein kinase (MAPK) pathways^36–40^, thus partially mimicking early T cell differentiation processes. Here, we show that signals induced by the PMA and ionomycin stimulation of P5424 cells resemble part, but not all the signals required for the β-selection process and aspects of subsequent T-cell maturation/activation, including the induction of the anti-apoptotic gene *Bcl2*. By combining transcriptomic and epigenomic experiments, we identified a set of lncRNAs, including many previously uncharacterized transcripts, associated with the regulation of key T-cell genes. Genetic inactivation of the PMA/ionomycin inducible lncRNA *XLOC_000895* (*Robnr*), located downstream of the *Bcl2* gene, resulted in impaired *Bcl2* activation, thus revealing a critical regulator of the *Bcl2* locus and highlighting the usefulness of the P5424 pro-T-cell line to dissect the molecular basis of T-cell regulatory networks.

## Results

### Effect of the PMA/ionomycin treatment on P5424 gene expression

The P5424 cell line was derived from DN thymocytes of *p53* and *Rag1* double knock-out mice^34^. Akin other DN-derived leukemic cell lines, the P5424 cells express the CD4 and CD8 surface markers, likewise double positive (DP) thymocytes^34,35^. However, these cells have a transcription signature similar to double negative (DN) thymocytes, which includes high expression of *Ptcra* and the Notch1-target gene *Hes1*, as well as, very low level of *Tcra* expression (Supplementary Fig. 1A,B). These observations suggest that P5424 cells are somehow blocked between the DN-to-DP transition during the β-selection process.

To study the gene regulatory networks downstream of the (pre-)TCR signaling during early T-cell differentiation we used a combination of PMA and ionomycin to stimulate the protein kinase C (PKC)- and the calcineurin-mediated pathways^36,41^ in the mouse P5424 T-cell precursor cell line. PMA/ionomycin treatment of early T-cell precursors has been shown to activate the pre-TCR signaling pathway and to induce the expression of the *Tcra* locus^37^. Based on the expression level of the *Tcra* gene, we determined that treatment with 10 ng/ml of PMA and 0.5 µg/ml of ionomycin for 4 h resulted in the highest gene induction (Supplementary Fig. 1A). Thus, we decided to use these conditions in further experiments. The PMA/ionomycin stimulation of P5424 cells reflects the β-selection by repressing the expression of the early T-cell markers *Ptcra* and *Hes1* and inducing the *Tcra* and *Egr1* genes (Supplementary Fig. 1B). To further validate these findings, we analyzed the expression of the human (h)CD25 in a stable transfected P5424 cell line, where hCD25 is under the control of the mouse *Ptcra* promoter^42^ (Supplementary Fig. 1C). As expected, the PMA/ionomycin stimulation caused an homogeneous loss of hCD25 expression at the surface of the P5424 cells (Supplementary Fig. 1D), meaning that the *Ptcra* promoter was strongly repressed by the PMA/ionomycin treatment.

The β-selection process has been shown to result in cell proliferation *in vivo*^1^. However, after the PMA/ionomycin treatment of P5424 cells, we observed a blockage of cell proliferation (Supplementary Fig. 1E), accompanied by an increased apoptosis compared to unstimulated cells (Supplementary Fig. 1G–F). This was consistent with previous results^43^ and might reflect an overstimulation of the cells, reminiscent of the negative selection occurring at later stages of thymic T cell maturation, which is mediated by apoptosis induced by strong T-cell stimuli^44^.

**Figure 1:**
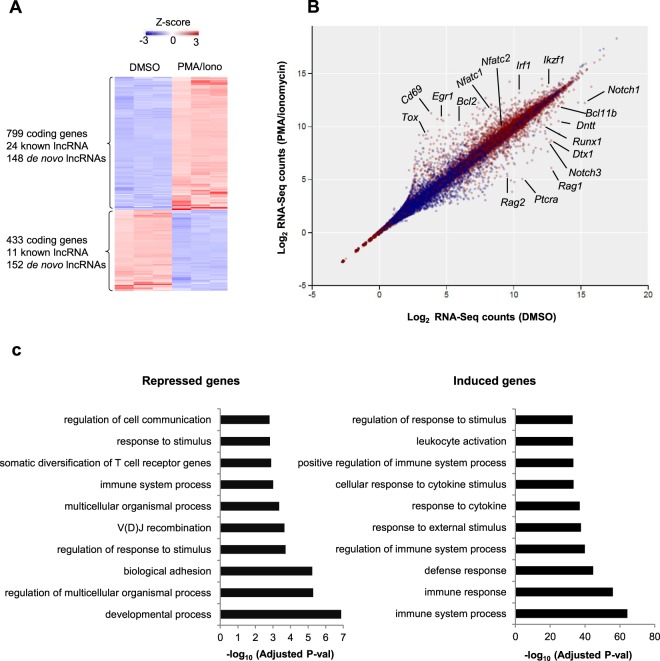
Effect of the PMA/ionomycin treatment on P5424 gene expression. (**A**) Heatmap showing the normalized RNA-seq signal of differentially expressed coding and lncRNA genes in 3 controls (DMSO) and 3 PMA/ionomycin-treated (PMA/Iono) P5424 cells. (**B**) Scatter plot highlighting upregulated and downregulated coding genes (red) and lncRNAs (blue) in response to PMA/ionomycin stimulation on P5424 cells. (**C**) The top 10 biological processes identified by functional enrichment analysis (g:Profiler) of coding genes that were affected by stimulation with PMA/ionomycin.

To have a comprehensive view of the transcriptional and epigenomics effects of the PMA/ionomycin treatment, we performed RNA-seq and ChIP-seq (for H3K4me3 and H3K27ac) experiments on mock (DMSO) and PMA/ionomycin-treated P5424 cells. Analysis of the RNA-seq for *de novo* discovery of lncRNAs identified 7098 transcripts corresponding to 6487 *de novo* lncRNA genes (Supplementary Dataset 1). As expected, most *de novo* lncRNAs were T-cell specifics (Supplementary Fig. 2A). The PMA/ionomycin treatment led to **799** induced and **433** repressed coding genes, as well as **172** induced and **163** repressed lncRNAs (including **148** and **152** *de novo* lncRNAs, respectively) (adjusted p-value < 0.01; fold change > 2; Supplementary Dataset 2; Fig. 1A). However, we did not observe substantial changes in the level of histone modifications at promoters of differentially regulated genes (data not shown). Visual inspection of significantly regulated genes reveals that several genes related to the early differentiation of T lymphocytes were significantly repressed (*e*.*g*., *Rag1/2*, *Ptcra*, *Dntt*, *Notch1*, *Notch3*, *Dtx1*) while genes associated with T-cell activation or maturation were induced (*e*.*g*., *Nfatc1*, *Nfatc2*, *Tox*, *Ikzf1*, *Cd*6*9*, *Egr1*) (Fig. 1B). Accordingly, functional enrichment analysis revealed that the repressed genes were related to development and early differentiation of T lymphocytes (e.g., developmental process, V(D)J recombination, somatic diversification of T-cell receptor genes), whereas the induced genes were linked to T-cell activation (e.g., immune response, response to cytokine, leukocyte activation) (Fig. 1C).

To precisely assess to what extent the PMA/ionomycin stimulation of P5424 cells resembles the β-selection process, we analyzed the relative enrichment of the β-selection signature obtained by comparing gene expression between DN4 and DN3a thymocytes^45^. As shown in Supplementary Figure 1H–I, genes repressed by the β-selection were enriched in unstimulated P5424 cells (Supplementary Fig. 1H), while β-selection induced genes were enriched in PMA/ionomycin treated cells (Supplementary Fig. 1I). However, we also noted that the expression of several β-selection induced (such as *Rorc*, *Ikzf3 and Camk4*, *Lef1*, *Nfatc3*) and repressed (*Lfng*, *Maml3*, *Dtx3/3 l*, *Il2ra* and *IL7r*) genes where not affected by the PMA/ionomycin treatment or regulated in the opposite way, suggesting that an incomplete transcriptional program was induced following PMA/ionomycin treatment. Thus, although the PMA/ionomycin stimulation of P5424 cells did not exactly match the β-selection process, it provides a good *in vitro* model for the analysis of mechanisms leading to early T-cell differentiation and activation.

### Functional annotation of PMA/ionomycin-regulated lncRNAs

**Figure 2:**
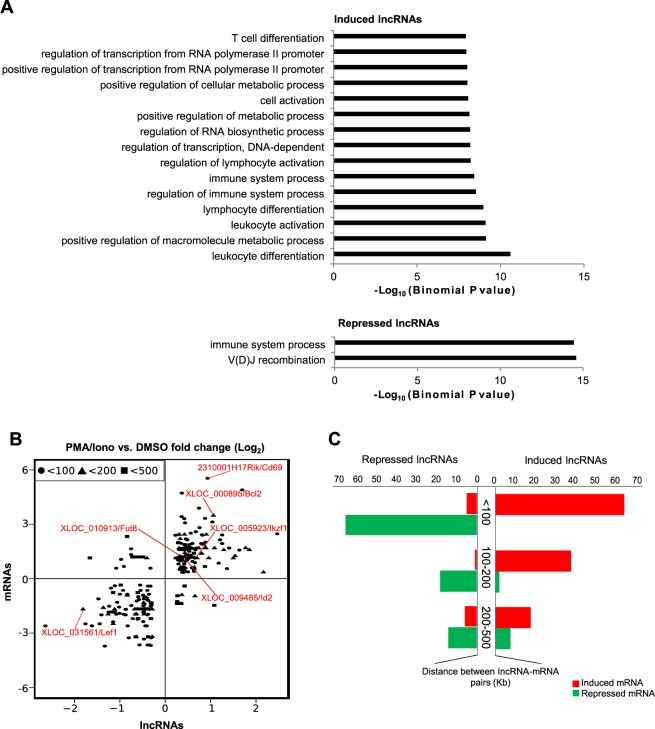
Correlation between lncRNAs and mRNAs regulation in P5424 cells. (**A**) The functional annotation (biological processes) of coding genes which are associated with PMA/ionomycin-regulated lncRNAs, using the GREAT tool. The top 15 most significant terms are indicated. The inverted Log10 of the Binomial P value is represented. (**B**) Scatter plot depicting the Log2 (fold-change between PMA/ionomycin and DMSO) of regulated mRNA/lncRNA pairs in respect to the distance between their TSSs (Kb). Pairs separated by less than 100 kb, 200 kb and 500 Kb are displayed. Typical examples are highlighted in red. (**C**) Bar plot displaying the number of regulated lncRNAs (x-axis) according to the expression state (induced/repressed) of their closest mRNA and in terms of the distance between them (y-axis).

As an initial assessment of the biological functions of lncRNAs affected by the PMA/ionomycin treatment, we investigated the biological processes enriched in the set of coding genes surrounding the differentially expressed lncRNAs, using the GREAT tool^46^. This tool assigns biological meaning to a set of non-coding genomic regions (in this case, the lncRNA loci) by analyzing the annotations of the surrounding genes using a binomial test. Strikingly, PMA/ionomycin-regulated lncRNAs were significantly associated with coding genes involved in the development and activation of T cells (e.g. V(D)J recombination and regulation of T lymphocyte differentiation) (Fig. 2A).

To determine whether there was a statistical association between the regulated lncRNAs and their neighboring mRNAs, we computed the distance between each induced or repressed lncRNA and the closest regulated mRNA, defined as lncRNA/mRNA pairs (Supplementary Dataset 3). We observed that pairs of lncRNA/mRNA separated by less than 500 kb were similarly co-regulated (Fig. 2B,C). Moreover, the lower the distance were between the two genes, the higher was the frequency of concordant changes (Fig. 2C). Indeed, we found that 40.74% of the regulated lncRNAs were located at less than 100 kb from a similarly regulated mRNA (Fig. 2C), demonstrating a significant association between consistently regulated lncRNA/mRNA pairs (P = 2e^−19^ and 1e^−30^ for the induced and repressed lncRNA/mRNA pairs located at less than 100 kb, respectively; hypergeometric test). However, no significant association was found between pairs of dissimilarly regulated lncRNAs and mRNAs, indicating that PMA/ionomycin regulated lncRNA/mRNA pairs are generally positively correlated. Overall, these results suggested that some of the PMA/ionomycin-regulated lncRNAs, or the associated regulatory sequences, might play a role in the *cis*-regulation of coding genes involved in T-cell differentiation or activation. This is consistent with previous studies highlighting that lncRNAs at various stages of the murine T lymphocytes development and differentiation are preferentially located adjacent to genes coding for cytokines or linage-specific transcription factors^18,20,47^.

### Validation of correlated lncRNA/mRNA pairs

**Figure 3:**
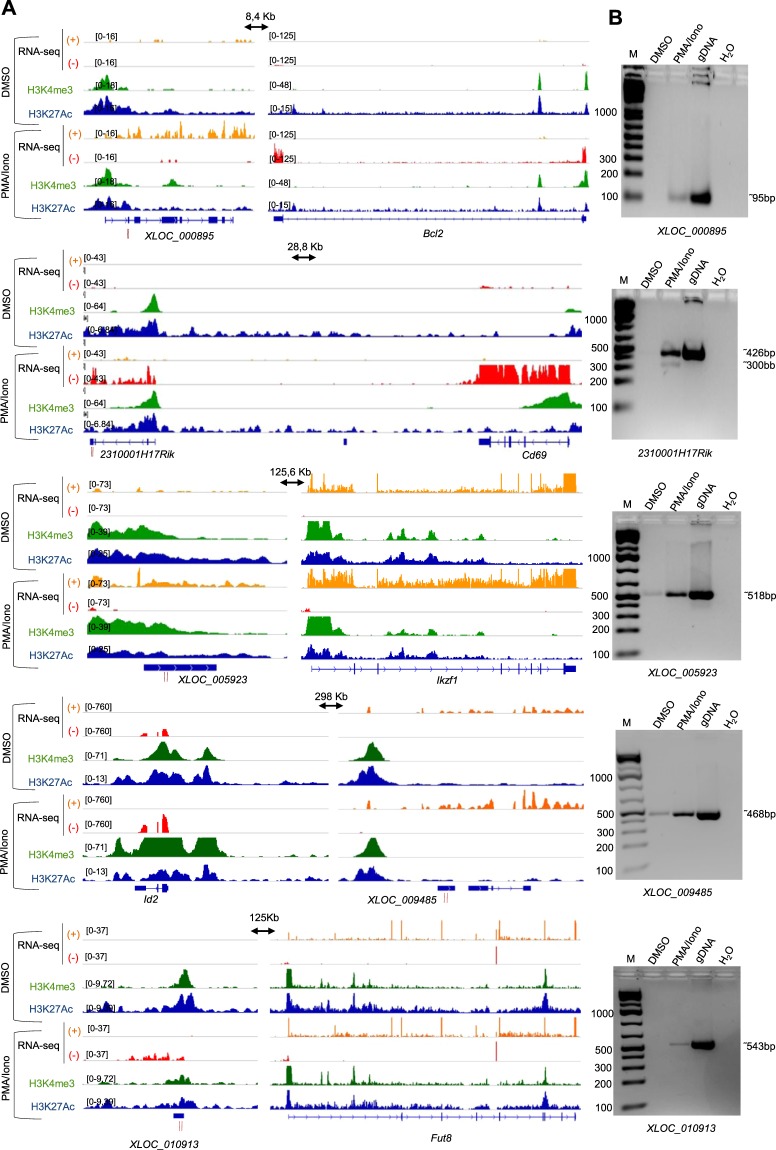
Selected co-induced lncRNA/mRNA pairs. (**A**) The genome browser screenshots of control cells (DMSO, upper panels) and PMA/ionomycin-treated cells (PMA/Iono, lower panels) show the RNA-seq data (strand + in yellow and strand − in red) as well as the ChIP-seq data (H3K4me3 in green and H3K27ac in blue) on the induced lncRNA loci and their nearby coding genes. Black arrows indicate the distance between the lncRNA and its neighboring gene. The primers used for RT-PCR validation are denoted by vertical red lines under the lncRNA position. (**B**) The PCR amplification of some lncRNAs that are induced by the PMA/ionomycin treatment are shown in control cells (DMSO), PMA/ionomycin-treated P5424 cells (PMA/Iono) and genomic DNA (gDNA). The *Actb* gene was used as an internal control in the experiments (Supplementary Fig. 3C).

**Figure 4:**
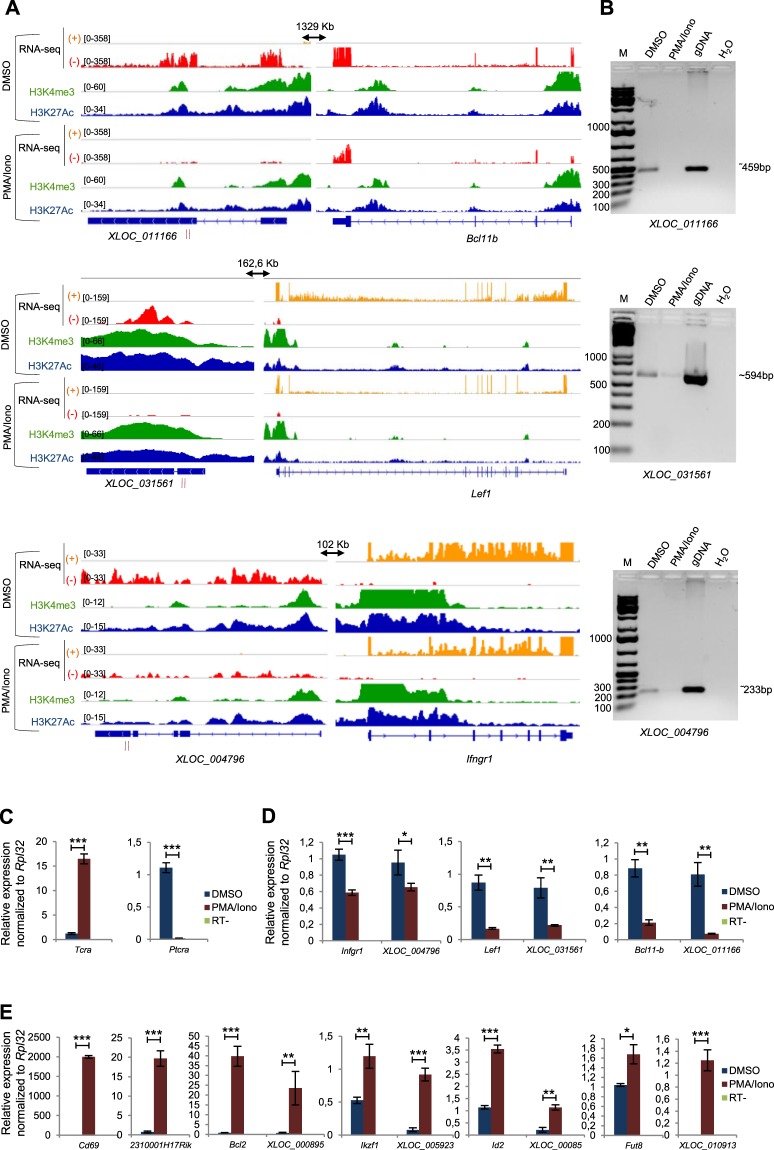
Selected co-repressed lncRNA/mRNA pairs. (**A**) The genome browser screenshots of control cells (DMSO, upper panels) and PMA/ionomycin-treated cells (PMA/Iono, lower panels) show the RNA-seq data (strand + in yellow and strand − in red) as well as the ChIP-seq data (H3K4me3 in green and H3K27ac in blue) on the repressed lncRNA loci and their nearby coding genes. Arrows indicate the distance between the lncRNA and its neighboring gene. The primers used for RT-PCR validation are denoted by vertical red lines under the lncRNA position. (**B**) The PCR amplification of some lncRNAs that are repressed by the PMA/ionomycin treatment are shown in control cells (DMSO), PMA/ionomycin-treated P5424 cells (PMA/Iono) and genomic DNA (gDNA). The *Actb* gene was used as an internal control in the experiments (Supplementary Fig. 3C). (**C**–**E**) RT-qPCR analyses in P5424 cells treated with DMSO or PMA/ionomycin of *Tcra* and *Ptcra* control genes (**C**), repressed lncRNA/mRNA pairs (**D**) and induced lncRNA/mRNA pairs (**E**). Gene expression was normalized to *Rpl32*. Statistical significance was assessed by Student’s *t*-test (unpaired, two-tailed) from 3 biological replicates (***P < 0.001, **P < 0.01, *P < 0.1). Data are represented with standard deviation.

The above observations pointed us to look more in detail for pairs of coregulated lncRNA/mRNA loci that might be involved in T-cell functions (Supplementary Dataset 3). We used H3K4me3 and H3K27ac ChIP-seq data to better map the 5′ regulatory regions of the lncRNA candidates. Visual inspection of the PMA/ionomycin-regulated loci led to the selection of 5 co-induced and 3 co-repressed lncRNA/coding gene pairs showing correlated expression and histone modification dynamics (Figs 3A, 4A, respectively). RT-PCR assessment of the selected lncRNAs retrieved the expected results (Figs 3B, 4B; Supplementary Fig. 3C and data not shown). Subsequently, we validated the co-regulation of the lncRNA/mRNA pairs in PMA/ionomycin- and DMSO-treated P5424 cells by RT-qPCR (Fig. 4D,E). As anticipated, selected loci were significantly co-regulated in the expected direction upon PMA/ionomycin treatment.

**Figure 5:**
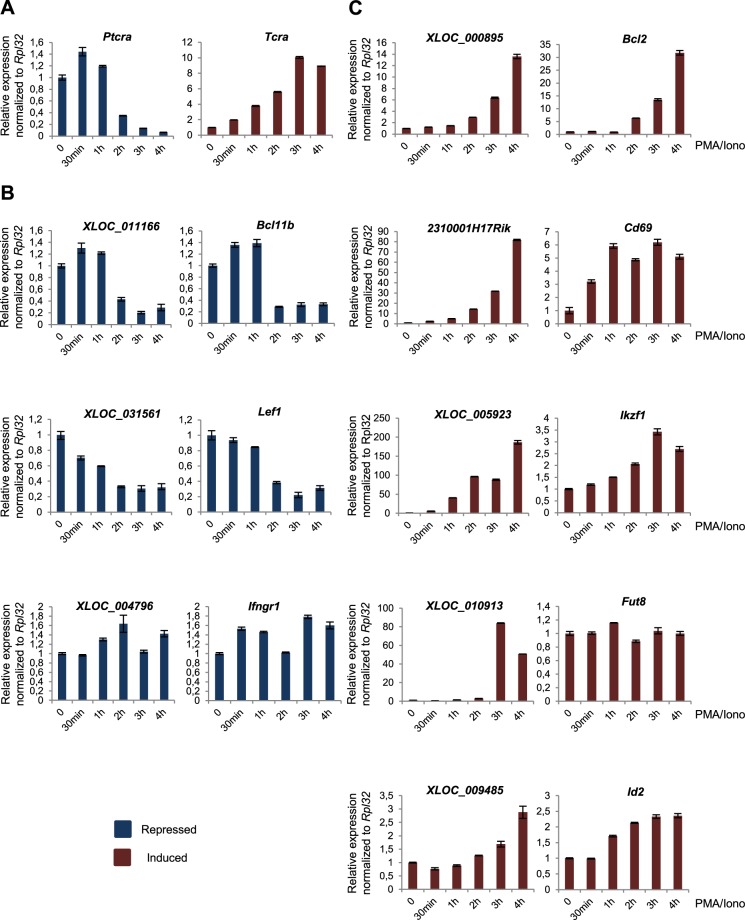
Kinetic studies of the lncRNA/mRNA pairs regulation upon PMA/ionomycin stimulation. **A**–**C**) P5424 cells were stimulated by PMA/ionomycin and collected at the indicated time points for RT-qPCR analysis of *Tcra* and *Ptcra* control genes (**A**), repressed lncRNA/mRNA pairs (**B**) and induced lncRNA/mRNA pairs (**C**). Experiments were performed in triplicates and the expression was normalized to *Rpl32*. Data are represented with standard deviation.

To further elucidate the relationships between the lncRNAs and their neighboring coding genes, as well as the potential *cis*-regulatory functions of these lncRNAs, we analyzed their gene expression kinetics after PMA/ionomycin stimulation. We collected PMA/ionomycin stimulated P5424 cells at different time points (0–30min-1h-2h-3h-4h) and performed RT-qPCR analysis. The progressive *Tcra* activation and *Ptcra* repression confirmed the quality of the kinetic experiments (Fig. 5A). Repression of the lncRNAs *XLOC_031561* and *XLOC_011166* expressions correlated well with the repression of their associated coding genes *Lef1* and *Bcl11b*, respectively (Fig. 5B). Likewise, some induced lncRNA/mRNA pairs showed a consistent regulation (*XLOC_000895/Bcl2* and *XLOC_005923/Ikzf1*), while the other induced lncRNA/mRNA pairs were less well correlated (Fig. 5C). Among the well correlated pairs, we found that the *XLOC_000895/Bcl2* pair displayed a highly similar kinetic of activation, hence strongly supporting a functional *cis*-regulatory link.

*Bcl2* is a major regulator of cell death (apoptosis) and is involved in T-cell mediated immune response, as well as in oncogenic transformation of lymphoid lineages^48–51^. The *XLOC_000895*/*Bcl2* locus was expressed at low levels in developing thymocytes as observed with our or published RNA-seq datasets (data not shown and Supplementary Fig. 4). As *Bcl2* is required for normal T-cell differentiation and maturation^52^, we hypothesized that this locus might be expressed at high level in a small population of thymocytes or at later T-cell maturation stages. Consistent with this, *Bcl2* has been shown to be up-regulated in DP thymocytes treated with PMA/ionomycin, likely reflecting cell survival during positive selection of DP cells^53^. To explore whether the *XLOC_000895*/*Bcl2* pairs is expressed at later stages of T-cell maturation or during T-cell activation, we reanalyzed a published RNA-seq dataset covering most stages of thymic and peripheral T-cell differentiation^18^. Interestingly, we observed that expression of the *XLOC_000895* and *Bcl2* genes were well correlated throughout T-cell differentiation (Supplementary Fig. 4A,B), with the highest expression of both genes in differentiated Th2 cell populations (Supplementary Fig. 4C). This suggests a potential role of this lncRNA locus in the regulation of *Bcl2* expression during T-cell maturation.

The *Bcl2* gene is often up-regulated in T-cell leukemia and lymphoma^54–56^. A recent study using a mouse model of T acute lymphoblastic leukemia (T-ALL) identified *Bcl2* as a direct target of TLX1 and NUP214-ABL1 oncogenes when both oncogenes were expressed in the same cells and suggested to be a potential therapeutic target in this context^47^. New analyses of this data demonstrated that the *XLOC_000895* lncRNA was also induced in TLX1/NUP214-ABL1 expressing thymocytes, similarly to *Bcl2* (Supplementary Fig. 5). Moreover, we observed an increase of H3K4 methylation at the promoter of *XLOC_000895* along with binding of TLX1 and STAT5 (a direct target of NUP214-ABL1 fusion protein) as well as p300 and BRD4 co-factors in TLX1/NUP214-ABL1 positive thymocytes (Supplementary Fig. 5E). These observations strongly suggested that *XLOC_000895* is a direct target in T-ALL and a potential regulator of *Bcl2*.

### *XLOC_000895* locus regulates the expression of *Bcl2* in P5424 cells

**Figure 6:**
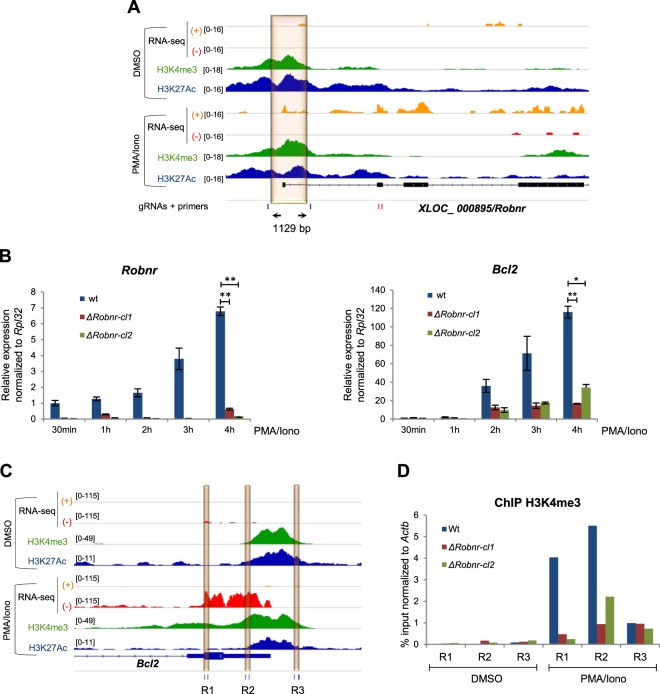
The *XLOC_000895*/*Robnr* locus regulates *Bcl2* expression. (**A**) Genome browser tracks showing the RNA-seq and ChIP-seq data on the *XLOC_000895*/*Robnr* locus. The CRISPR/Cas9 targeted region is highlighted in orange. The location of guide RNAs (gRNAs) are denoted by vertical blue lines and the size of the targeted region is indicated. The primers used for RT-qPCR analysis are denoted by vertical red lines. (**B**) Relative expression levels of *Bcl2* and *Robnr* at different time points of the PMA/ionomycin treatment in wild-type (wt) and *Robnr*-mutated clones, Δ*Robnr-cl1* and Δ*Robnr-cl2*. *Rpl32* was used for normalization. The statistical significance was assessed at 4 h of stimulation from 3 biological replicates by a Student’s *t*-test (unpaired, one-tailed; ***P < 0.001, **P < 0.01, *P < 0.1). Data are represented with standard deviation. Details about the gRNA sequences, the PCR primers and the expected amplicons are provided in Supplementary Table 1. (**C**) Genome browser tracks showing the RNA-seq and ChIP-seq data surrounding the *Bcl2* promoter. Three genomic regions displaying a higher H3K4me3 enrichment in PMA/ionomycin stimulated cells are highlighted. The primers used for ChIP validation are denoted by vertical blue lines. (**D**) ChIP-qPCR analysis of the H3K4me3 enrichment on the *Bcl2* promoter in wild-type (wt) and the two *Robnr*-mutated clones, after an exposure to DMSO or PMA/ionomycin (PMA/Iono) for 4 h. The graph shows the results of 3 replicates. The promoter of *Actb* was used for normalization.

We named the *XLOC_000895* lncRNA *Robnr* (*Regulator of Bcl2 non-coding RNA*). The *Robnr* lncRNA has 3 predicted isoforms sharing a single transcription start site and 8 exons (Supplementary Dataset 1). To investigate whether the *Robnr* locus plays a direct role in the regulation of *Bcl2* expression, we deleted its promoter and the 5′ region of this lncRNA using CRISPR/Cas9-mediated genome editing (Fig. 6A). Two homozygous knockout clones were identified by PCR screen (Δ*Robnr-cl1* and Δ*Robnr-cl2;* Supplementary Fig. 6). Next, the expression of *Bcl2* gene was assessed by RT-qPCR at different time points of PMA/ionomycin stimulation in *Robnr* deleted cells (Fig. 6B). As expected, the expression of *Robnr* was reduced to background levels in the two Δ*Robnr* clones, demonstrating the full inactivation of the lncRNA gene. Interestingly, the induction of *Bcl2* upon PMA/ionomycin treatment was significantly impaired in the two Δ*Robnr* clones (Fig. 6B). To assess whether *Robnr* influences the epigenetic regulation of the *Bcl2* locus, we analyzed the enrichment of H3K4me3 by ChIP-qPCR on three dynamic regions of the *Bcl2* promoter (Fig. 6C,D). Accordingly, we observed a substantial reduction of H3K4me3 enrichment at the two most downstream regions of the *Bcl2* promoter in the Δ*Robnr* clones upon PMA/ionomycin treatment (Fig. 6D). Taken together, our data strongly support the involvement of *Robnr*, or its associated regulatory sequences, in the regulation of the pro-apoptotic *Bcl2* gene.

## Discussion

T-cell differentiation is subjected to several developmental checkpoints which are controlled by either pre-TCR or TCR complexes. In particular, (pre-)TCR downstream signaling triggers complex intracellular pathways resulting in wide changes in the transcriptional and epigenetic program of genes associated with cell survival, proliferation and differentiation^4^. However, a precise understanding of the regulatory mechanisms at play during T-cell differentiation is still lacking. Defining the players which allow T cells to pass from immature stages to an activated mature stage and their gene targets may not only help to gain a better understanding of the mechanisms underlying the immune response regulation, but also provide insight into disease states and thereby facilitate the identification of new therapeutic targets in which T-cell disorders play pathogenetic roles.

Current T cell differentiation models have limitations due to growth defect, transfection inefficiency, feeders requirement for co-culture or presence of undefined abnormalities because they are derived from natural tumor blasts^37,57^. Here, we showed that stimulation of P5424 cells by PMA/ionomycin roughly mimics the β-selection process from pro-T cells like thymocytes, as well as aspects of subsequent T cell maturation/activation, including *Bcl2* up-regulation. This system will allow a better dissection of the kinetic events triggered by the (pre-)TCR signaling that might be missed in sorted primary T cell precursors from steady state populations. This study also confirmed that integration of Ca^2+^ and kinase signaling is sufficient for efficient activation of inducible gene expression in T-cell precursors.

Although PKC activation and calcium elevation may be part of the biochemical signals induced by (pre-)TCR signaling during early T cell differentiation, signals induced by PMA and ionomycin resemble part, but not all, of the signals required for the β-selection and might also reflect subsequent T-cell maturation and activation steps. Indeed, several differences suggested that an incomplete transcriptional program is induced by the PMA/ionomycin treatment of P5424 cells. On the one hand, not all the genes regulated by the β-selection process were regulated by the PMA/ionomycin signaling or were not regulated in the same way (Supplementary Fig. 1H-,I). On the other hand, the β-selection induces cell proliferation^45,58^, while PMA/ionomycin treatment of the P5424 cells resulted in a blockage of cell proliferation and an induction of apoptosis (Supplementary Fig. 1E,F). More generally, in Jurkat cells, TCR stimulation with various concentrations of anti-CD3 resulted in digital activation of Nf-κB^59,60^, which is a known regulator of the β-selection^61^. In contrast, treatment of Jurkat cells with various concentration of PMA/ionomycin resulted in analog type of activation^59^. Thus, stimulation of T cell lines by PMA/ionomycin might mimic some, but not all, the signals received during T cell differentiation.

Scientific evidences accumulated over the last decade demonstrated a complex involvement and diverse activities of non-coding RNA in the regulation of gene expression^11–13^. However, few studies concerning their roles in the development, differentiation and/or activation of T lymphocytes are available^62^. In our study, we used the P5424 cellular model to investigate the lncRNAs involvement in T lymphocytes development. Using RNA-seq data of P5424 cells stimulated with PMA/ionomycin, we identified 163 repressed and 172 induced lncRNAs (Fig. 1A,B), with the vast majority representing de novo transcripts. Interestingly, lncRNAs regulated by the PMA/ionomycin stimulation were found to be close to genes involved in T-cell development (Fig. 2A). In addition, we observed a positive co-regulation of neighboring lncRNAs and coding genes (Fig. 4D,E). This finding is consistent with the emerging roles of lncRNAs in the modulation of expression of their nearby protein-coding genes^63^. For instances, functional screening have suggested that approximately 10% of the lncRNA promoters may regulate nearby genes^64,65^. Several examples from the hematopoietic system have demonstrated specific *cis*-regulation by lncRNAs, including *Bcl11b*^20^, *IGF1R*^28^, *Ifng*^23^, *y-globin*^66^ or *MYC*^67^ loci. However, the underlying cis-regulatory mechanisms are not yet fully understood^68^, and might require the transcript itself, the transcriptional process or the associated *cis*-regulatory elements (either enhancers or promoters)^63,69,70^.

As a proof of principle, we assessed the role of *XLOC_000895* (*Robnr*) lncRNA, located downstream of the *Bcl2* gene (8.4 kb from the end of *Bcl2*). Both genes were highly induced after PMA/ionomycin-treatment of the P5424 cells and their kinetics of induction were tightly correlated. The induction of the pro-survival gene *Bcl2* was contrasting with the high level of apoptosis observed following PMA/ionomycin treatment, and might reflect an overstimulation of the T cell line^43^. Genetic deletion of *Robnr* using the CRISPR/Cas9 editing significantly reduced the induction of *Bcl2* upon PMA/ionomycin stimulation (Fig. 6), implying that the *Robnr* locus is a critical regulator of *Bcl2* expression and the epigenetic marking of the *Bcl2* promoter. While it is possible that the *Robnr* lncRNA transcript directly promotes *Bcl2* transcription, our current data did not exclude the possibility that *Robnr* deletion inactivated an enhancer required for normal *Bcl2* transcription. The precise mechanism(s) leading to *Bcl2* regulation by the *Robnr* locus, as well as the specific contribution of the *Robnr* transcript and/or its associated regulatory elements should be addressed in the future.

T-cell homeostasis requires maintaining a delicate balance between the rates of cell death (apoptosis) and cell proliferation. Some of the most important genes controlling cell death belong to the Bcl-2 superfamily. These proteins are known to determine whether developing T cells undergo apoptosis in the thymus or survive to reach peripheral organs^50,71–74^. *Bcl2* deficient mice demonstrate normal differentiation of both B and T lineages, but these mice fail to maintain lymphoid homeostasis and display lymphocytes apoptosis in response to activation stimuli^75^. Previous studies have also suggested that *Bcl2* is not induced during β-selection, whereas *Bcl2-A1* (Bcl2-related protein A1) is likely to be an important mediator of thymocyte survival during this process^76^. However, *Bcl2-A1* is not expressed in P5424 cells in neither unstimulated or stimulated conditions. p53 is required for apoptosis induced by DNA damage in thymocytes^77–79^ and also for the deletion of cells that fail to progress through the β-selection checkpoint^80^. The absence of p53 gene in P5424 cells could therefore bias the signals observed upon PMA/ionomycin stimulation. While expression of Bcl2-A1 and repression of p53 are well recognized to control cell survival downstream from the pre-TCR, it has also been reported that overexpression of *Bcl2*, and to a minor extend of *Bcl2-A1*, rescued the survival defect of NFAT5-deficient thymocytes observed during β-selection^81^. This suggests that *Bcl2* could also be relevant in the pro-survival function of the pre-TCR signaling. Besides its potential role in the β-selection, the regulation of *Bcl2* by the *Robnr* locus might be important at other stages of T cell differentiation or maturation. For instances, co-activation of *Robnr* and *Bcl2* is observed during Th2 differentiation (Supplementary Fig. 4C) pointing out potential implications of this lncRNA in the immune responses. *Bcl2* has been shown to be up-regulated in DP thymocytes treated with PMA/ionomycin, likely reflecting cell survival during positive selection of DP cells^53^. Whether *Robnr* is also induced in cells undergoing positive selection need to be investigated in the future.

Evasion of apoptosis is a hallmark of human cancer and is often mediated by overexpression of the pro-survival Bcl-2 family proteins^82^. Defective signaling through the Bcl-2 family would enable thymocytes flagged for destruction to survive and acquire additional lesions that promote full malignant transformation. This leads T-ALL blasts to become dependent on this specific pathway whose actions perturb the normal balance between thymocyte life and death signaling cues^83^. Indeed, *Bcl2* overexpression can act as a major determinant of chemotherapy resistance^84^. Therefore, the pro-survival members of the Bcl-2 family as well as the pathways that signal upstream of these proteins are attractive candidate targets and *Bcl2* inhibitors have been proposed as a novel therapeutic strategy in T-ALL^85,86^. Strikingly, *Robnr* and *Bcl2* are co-induced in a mouse model of (*Bcl2*-dependent) T-ALL (Supplementary Fig. 5)^47^. Although, we did not detect the expression of *Robnr* in human T-ALL cell lines (data not shown), the human orthologous region of the *Robnr* promoter appears to be in an open chromatin configuration^87^. Strikingly, this region was suggested to be involved in Notch-dependent chemoresistance in T-ALL and can be specifically targeted by the use of epigenetic inhibitors that repressed the *Bcl2* expression^87^.

In conclusion, using a model system that approximates early T cell development, we identified lncRNAs potentially regulating T cell development and functions. This *in vitro* model system should allow us to better understand the kinetics of transcriptional regulation events driven by (pre-)TCR signaling that might be missed by studying steady state populations *ex vivo*. The specific contribution of the *Robnr* locus to the progression of leukemia and/or T cell function and development through the regulation of apoptosis is an important outcome of this work that should be explored in the future.

## Materials and Methods

### Cell culture

The P5424 line was grown in RPMI-1640 GlutaMAX ™ medium (Thermo Fisher Scientific, 61870-010), supplemented with 10% fetal calf serum (Thermo Fisher Scientific, 10270-106) at 37 °C, 5% CO_2_. Cells were subcultured every 2–3 days and routinely tested for mycoplasma contamination.

### Stimulation of P5424 cells

P5424 cells were grown in 6-well plates at density of 3 × 10^5^ cells/ml. Cells were treated by DMSO or PMA at 10 ng/ml (P1585, Sigma) and ionomycin at 0.5 μg/ml (I3909, Sigma) for 4 hours in triplicates, except for experiments described in Supplementary Figure 1. In the kinetic studies, cells were stimulated as described above, but they were collected at the indicated time points after treatment.

### Cell proliferation and apoptosis assays

P5424 cells were treated with DMSO or PMA/ionomycin for 4 hours. Cells were washed and recovered for 24, 48 or 72 hours. The cell proliferation was analyzed with the Scepter™ cell counter (Millipore) and a 60 μm sensor tip, following the manufacturer instructions. To quantify apoptotic cells, the samples were washed with phosphate-buffered solution (PBS), then stained with propidium iodide (PI) and Annexin-V using the Annexin-V-FLUOS staining kit (11858777001, Roche) following the manufacturer instructions. Cells stained with Annexin-V-FLUOS and PI were detected by flow cytometry analysis (FACS) using a LSRII cytometer (BD Biosciences). FlowJo 7.6.5 was used to analyze the FACS data. The percentage of PI and Annexin-V positive cells was calculated.

### RNA extraction and cDNA synthesis

The extraction of total RNA was performed using the RNeasy Plus Mini kit (Qiagen) according to the protocol recommended by the supplier. Total RNA was quantified using a Nanodrop 1000 spectrophotometer (Thermo Scientific) and stored at −80 °C until needed. The reverse transcription was carried out using the Invitrogen™ SuperScript™ VILO™ Master Mix according to the manufacturer instructions.

### RNA-seq

Poly(A) RNA was isolated from three replicates of P5424 cells treated with DMSO or PMA/ionomycin and was used for the RNA-seq library preparation, using the TruSeq RNA Library Prep Kit v2 (Illumina). Libraries were paired-end sequenced on the Illumina NextSeq 500 sequencer. Reads were aligned using STAR aligner (v2.4.2a) with arguments “outFilterMismatchNoverLmax” and “outFilterMultimapNmax” set to 0.08 and 1, respectively. Transcripts discovery was performed using Cufflinks (v2.2.1) with the “library-type” argument set to fr-firstrand, and a GTF file obtained from GENCODE (“Comprehensive gene annotation”, vM1) provided as the genomic annotation. The GTF files produced for each sample by Cufflinks were combined using Cuffmerge. The “class code” assigned to each transcript by Cuffmerge was used to defined unknown transcripts (class code “u”). Only *de novo* transcripts with counts greater than 0 in at least one RNA-seq sample were kept for subsequent analyses (Supplementary Dataset 1). These *de novo* transcripts were combined with the GENCODE GTF file to produce the final genomic annotation that was provided to FeatureCounts (v1.4.6-p4) for quantification. Differential gene expression was performed using DESeq2 (v1.6.3). To create bigwig files, reads from Watson and Crick strands were selected using SAMtools (v0.1.9) and provided to the bam2wig.py script from the RSeQC program suite (v2.6.4) (Supplementary Dataset 2). Public RNA-seq data from Hu *et al*.^88^ and Vanden Bempt *et al*.^47^ were downloaded from the GEO database (accession numbers GSE48138 and GSE102209, respectively) and processed as described above. RNA-seq profiles were visualized using the IGV genome browser^89^.

### Tissue specificity

To estimate the bias toward tissue-specificity for each transcript, we processed a set of fastq files obtained from the SRA database (accession number SRP012040). This dataset corresponds to 30 mouse tissues sequenced with a paired-end strategy on an Illumina HiSeq2000^90^. After read mapping, gene expression levels were quantified using Cuffdiff ^91^. Based on the log2-transformed FPKM values, we assessed the tissue-specificity for each gene across every tissue using the tau score, as described by Kryuchkova-Mostacci and Robinson-Rechavi^92^. The tau score formula is:

$${\boldsymbol{Tau}}=\sum _{{\boldsymbol{i}}=0}^{{\boldsymbol{n}}}\frac{(1-\widehat{{{\boldsymbol{x}}}_{{\boldsymbol{i}}}})}{{\boldsymbol{n}}-1};\,\widehat{{{\boldsymbol{x}}}_{{\boldsymbol{i}}}}=\frac{{{\boldsymbol{x}}}_{{\boldsymbol{i}}}}{{\boldsymbol{ma}}{{\boldsymbol{x}}}_{1\le {\boldsymbol{i}}\le {\boldsymbol{n}}}({{\boldsymbol{x}}}_{{\boldsymbol{i}}})}$$

where: n corresponds to the number of samples, *X*_*i*_ to the expression level in condition i, and Max(x_i_) to the maximum expression level through all tissues.

### Genomic pairing of lncRNA and mRNA

For each differentially expressed lncRNA we identified the closest differentially expressed mRNA (hereafter lncRNA/mRNA pairs) and computed the distance between the two TSSs. Supplementary Dataset 3 provides the list of co-regulated lncRNA/mRNA pairs. All plots (bar and scatter) were done using the ggplot2 R package^93^.

### Functional enrichment analysis

Gene ontology (GO) enrichment of biological processes was assessed on PMA/ionomycin-regulated coding genes using the g:Profiler web server^94^. The top 10 GO terms enrichments having the lowest P value were used to select the annotations (Fig. 1C). Functional enrichment of biological processes associated with coding genes surrounding PMA/ionomycin-regulated lncRNAs was performed using the online tool GREAT (Genomic Regions Enrichment of Annotations Tool)^46^ and default setting. GREAT assigns each gene a regulatory domain consisting of a basal region of −5 kb/+1 kb from TSS, and an extension up to the nearest gene basal region, but no more than 1 Mb in both directions. Subsequently, it assigns biological meaning to a set of provided non-coding genomic regions (in this case, the lncRNA loci) by analyzing the annotations of the surrounding genes, using a Binomial test. We selected the first 15 significant GO terms with the lowest Binomial P values (Fig. 2A).

### Gene set enrichment analysis

To analyze the extent of concordance between the β-selection and the PMA/ionomycin stimulation process, we performed a Gene Set Enrichment Analysis (GSEA)^95,96^. For this purpose, we extracted a β-selection signature from the ‘immgen’ webtool (http://rstats.immgen.org/PopulationComparison) by comparing the gene expression between DN4 and DN3a thymocytes, based on the microarray data from Mingueneau *et al*.^45^. GSEA software was downloaded from http://software.broadinstitute.org/gsea/index.jsp.

### Qualitative analyses of gene expression

cDNAs from DMSO- or PMA/ionomycin-treated P5424 cells were analyzed by PCR with cycles of 95 °C for 1 minute, followed by 30 cycles including denaturation at 95 °C for 20 seconds, hybridization to Tm which varied from 60 °C to 64 °C, for 20 seconds and extension step at 68 °C for 1 minute. A final extension step at 68 °C for 4 minutes was performed. The 25 µl PCR reactions were prepared with 1 μl of cDNA from the RT-PCR reaction in accordance with the Herculase II Fusion protocol (Agilent, Waldbronn, Germany). The amplicons were analyzed by agarose gel electrophoresis by adjusting the percentage of agarose in respect of the expected fragments length. The PCR analysis of the lncRNAs regulated by the PMA/ionomycin stimulation was carried out using specific primers (Supplementary Table 1). Genomic DNA was used as an amplification control, *Egr1* and *Tcra* genes were used to check the stimulation efficiency and *Actb* gene was used as a sample quality control.

### Quantitative analyses of gene expression

The qPCR reactions were performed using the Applied Biosystems™ *QuantStudio*™ *6* Flex Real-Time PCR System with Power SYBR® Green PCR Master Mix. Primers sequences are listed in Supplementary Table 1. *Rpl32* was used as a reference gene to normalize the qPCR results. The Student’s t-test was performed (unpaired, two-tailed) from 3 biological replicates (***P < 0.001, **P < 0.01, *P < 0.1). Data are represented with standard deviation.

### Chromatin immunoprecipitation (ChIP)

A total of 5 × 10^6^ of DMSO or PMA/ionomycin treated P5424 cells was crosslinked with 1% formaldehyde for 10 min at 20 °C, followed by a quench with glycine at a final concentration of 250 mM. Pelleted cells were washed twice with ice-cold PBS, and then re-suspended in lysis buffer (20 mM Hepes pH 7.6, 1% SDS, 1X protease inhibitor cocktail) at final cell concentration of 15 × 10^6^ cells/ml. The chromatin was sonicated with a Bioruptor (Diagenode) to reach an average chromatin fragment length of 200–400 bp (5 pulses of 30 sec ON and 30 sec OFF). For each immunoprecipitation, an aliquot of sonicated cell lysate, equivalent to 5 × 10^5^ cells, was diluted with SDS-free dilution buffer. Specific antibodies and proteinase inhibitor cocktail were added to the lysate and mixed overnight at 4 °C. The antibodies used were the following: H3K4me3 (C15410003-50) and H3K27ac (C15410196, Diagenode). The next day, Protein A-coupled magnetic beads (Invitrogen) were washed twice with dilution buffer (0.15% SDS, 0,1% BSA), added to the lysate and placed on the rotating wheel for 1 hour at 4 °C. Beads were then washed with each of the following buffers: once with the Wash Buffer 1 (2 mM EDTA, 20 mM Tris pH 8, 1% Triton, 0.1% SDS, 150 mM NaCl), twice with the Wash Buffer 2 (2 mM EDTA, 20 mM Tris pH 8, 1% Triton, 0.1% SDS, 500 mM NaCl) and twice with the Wash Buffer 3 (1 mM EDTA, 10 mM Tris pH 8). Finally, beads were eluted in Elution buffer (1% SDS, 0.1 M NaHCO3) and mixed on a rotating wheel at room temperature for 20 min. To reverse the crosslink, 5 M NaCl and 10 mg/ml of proteinase K were added to the eluted material and to an input (10% of the sonicated chromatin used for immunoprecipitation). The samples were then incubated overnight at 65 °C. The next day, the DNA was purified with the QIAquick PCR Purification Kit (Qiagen) and eluted in 20 µl of water. Quantitative real-time PCR with SYBR green was used to quantify the immunoprecipitated DNA. The primers used are listed in Supplementary Table 1. Data represent the percentage of input normalized to the *Actb* promoter.

### ChIP-seq

ChIP-seq libraries for H3K4me3 and H3K27ac in P5424 cells treated with DMSO or PMA/ionomycin were generated with the MicroPlex Library Preparation Kit (Diagenode), according to the manufacturer instructions. The libraries were sequenced in paired-end 75/75nt mode using the NextSeq® 500/550 (Illumina), according to manufacturer’s instructions. Reads were mapped to the mm9 reference genome using standard procedures. ChIP-seq profiles were visualized using the IGV genome browser^89^.

### CRISPR-Cas9 genome editing

To knock-out the *XLOC_000895/Robnr lncRNA*, a gRNAs was designed on each side of the targeted region using the CRISPRdirect tool^97^. The gRNAs were cloned into a gRNA cloning vector (Addgene, 41824). Then 1 × 10^5^ cells were co-transfected with 5 μg of the hCas9 vector (Addgene, 41815) and 5 μg of each gRNA vector using the Neon Transfection System (Thermo Fisher Scientific). Two days later, cells were seeded in 96-well plates at limiting dilution (0.5 cells per well) for clonal expansion. Individual cell clones were screened after 10–14 days for homologous allele deletion by direct PCR using Phire Tissue Direct PCR Master Mix (Thermo Fisher Scientific) according to the manufacturer protocol. Forward and reverse primers were designed to bracket the targeted regions, allowing for the detection of knock-out or wild-type alleles. Clones were considered to have undergone homologous allele deletion if they showed the expected deletion band and no wild-type band on an agarose gel after PCR amplification. We obtained two clones with biallelic deletion (Supplementary Fig. 6). The PCR products corresponding to the deleted fragments were purified using the MinElute Purification kit (Qiagen) and sequenced (Eurofins Genomics) to determine the exact break points. Δ*Robnr*-cl1 had the expected 1.1 kb deletion (chr1:108412002–108413119), while Δ*Robnr*-cl2 had a deletion of 1.2 kb (chr1:108411914–108413135). Primers and gRNAs sequences are listed in Supplementary Table 1.

## Supplementary information

supplementals

Dataset 1

Dataset 2

Dataset 3

## Data Availability

RNA-seq and ChIP-seq data described in this study are available in GEO database under the accession number GSE120655 (http://www.ncbi.nlm.nih.gov/geo/).

## References

[1] Hayday AC, Pennington DJ (2007). Key factors in the organized chaos of early T cell development. Nat Immunol.

[2] Spicuglia S, Zacarias-Cabeza J, Pekowska A, Ferrier P (2010). Epigenetic regulation of antigen receptor gene rearrangement. F1000 Biology Reports.

[3] Taghon T, Rothenberg EV (2008). Molecular mechanisms that control mouse and human TCR-alphabeta and TCR-gammadelta T cell development. Semin Immunopathol.

[4] Carpenter AC, Bosselut R (2010). Decision checkpoints in the thymus. Nat Immunol.

[5] Pekowska A (2011). H3K4 tri-methylation provides an epigenetic signature of active enhancers. EMBO J.

[6] Zhang JA, Mortazavi A, Williams BA, Wold BJ, Rothenberg EV (2012). Dynamic transformations of genome-wide epigenetic marking and transcriptional control establish T cell identity. Cell.

[7] Aifantis I, Raetz E, Buonamici S (2008). Molecular pathogenesis of T-cell leukaemia and lymphoma. Nat Rev Immunol.

[8] Ntziachristos, P., Abdel-Wahab, O. & Aifantis, I. Emerging concepts of epigenetic dysregulation in hematological malignancies. *Nature Immunology* (2016). 10.1038/ni.3517 PMC5134743 27478938

[9] Notarangelo LD (2014). Immunodeficiency and immune dysregulation associated with proximal defects of T cell receptor signaling. Curr Opin Immunol.

[10] Spicuglia S, Maqbool MA, Puthier D, Andrau JC (2013). An update on recent methods applied for deciphering the diversity of the noncoding RNA genome structure and function. Methods.

[11] Geisler S, Coller J (2013). RNA in unexpected places: long non-coding RNA functions in diverse cellular contexts. Nat Rev Mol Cell Biol.

[12] Guttman M, Rinn JL (2012). Modular regulatory principles of large non-coding RNAs. Nature.

[13] Morceau F, Chateauvieux S, Gaigneaux A, Dicato M, Diederich M (2013). Long and short non-coding RNAs as regulators of hematopoietic differentiation. Int J Mol Sci.

[14] Bonasio R, Shiekhattar R (2014). Regulation of transcription by long noncoding RNAs. Annu Rev Genet.

[15] Alvarez-Dominguez JR (2014). HF. Global discovery of erythroid long noncoding RNAs reveals novel regulators of red cell maturation. blood.

[16] Atianand MK, Fitzgerald KA (2014). Long non-coding RNAs and control of gene expression in the immune system. Trends Mol Med.

[17] Fitzgerald KA, Caffrey DR (2014). Long noncoding RNAs in innate and adaptive immunity. Curr Opin Immunol.

[18] Zhu L, Xu PC (2013). Downregulated LncRNA-ANCR promotes osteoblast differentiation by targeting EZH2 and regulating Runx2 expression. Biochem Biophys Res Commun.

[19] Xia, F. *et al*. Dynamic Transcription of Long Non-Coding RNA Genes during CD4+ T Cell Development and Activation. *PLoS ONE* **9** (2014). 10.1371/journal.pone.0101588 PMC4086894 25003630

[20] Isoda T (2017). Non-coding Transcription Instructs Chromatin Folding and Compartmentalization to Dictate Enhancer-Promoter Communication and T Cell Fate. Cell.

[21] Pagani M (2013). Role of microRNAs and long‐non‐coding RNAs in CD4+ T‐cell differentiation. Immunological Reviews..

[22] Casero D (2015). Long non-coding RNA profiling of human lymphoid progenitor cells reveals transcriptional divergence of B cell and T cell lineages. Nature Immunology.

[23] Gomez JA (2013). The NeST long ncRNA controls microbial susceptibility and epigenetic activation of the interferon-gamma locus. Cell.

[24] Willingham AT (2005). A strategy for probing the function of noncoding RNAs finds a repressor of NFAT. Science.

[25] Wang Y (2015). Long noncoding RNA derived from CD244 signaling epigenetically controls CD8+ T-cell immune responses in tuberculosis infection. Proc Natl Acad Sci USA.

[26] Garzon R (2014). Expression and prognostic impact of lncRNAs in acute myeloid leukemia. Proc Natl Acad Sci USA.

[27] Yang, X. *et al*. A Network Based Method for Analysis of lncRNA-Disease Associations and Prediction of lncRNAs Implicated in Diseases. *PLOS ONE* **9** (2014). 10.1371/journal.pone.0087797 PMC3909255 24498199

[28] Trimarchi T (2014). Genome-wide mapping and characterization of Notch-regulated long noncoding RNAs in acute leukemia. Cell.

[29] Wallaert, A. *et al*. Long noncoding RNA signatures define oncogenic subtypes in T-cell acute lymphoblastic leukemia. *Leukemia* (2016). 10.1038/leu.2016.82 27168467

[30] Ngoc, P. C. T. *et al*. Identification of novel lncRNAs regulated by the TAL1 complex in T-cell acute lymphoblastic leukemia. *Leukemia* (2018). 10.1038/s41375-018-0110-4 PMC8197659 29654272

[31] Gioia R (2017). LncRNAs downregulated in childhood acute lymphoblastic leukemia modulate apoptosis, cell migration, and DNA damage response. Oncotarget.

[32] Alvarez-Dominguez JR, Lodish HF (2017). Emerging mechanisms of long noncoding RNA function during normal and malignant hematopoiesis. Blood.

[33] Salviano-Silva, A., Lobo-Alves, S. C., Almeida, R. C., Malheiros, D. & Petzl-Erler, M. L. Besides Pathology: Long Non-Coding RNA in Cell and Tissue Homeostasis. *Noncoding**RNA* **4** (2018). 10.3390/ncrna4010003 PMC5890390 29657300

[34] Mombaerts P, Terhorst C, Jacks T, Tonegawa S, Sancho J (1995). Characterization of immature thymocyte lines derived from T-cell receptor or recombination activating gene 1 and p53 double mutant mice. Immunology.

[35] Vanhille L (2015). High-throughput and quantitative assessment of enhancer activity in mammals by CapStarr-seq. Nat Commun.

[36] Chatila T, Silverman L, Millerand R, Geha R (1989). Mechanisms of T cell activation by the calcium ionophore ionomycin. J Immunol.

[37] Del Blanco B, Garcia-Mariscal A, Wiest DL, Hernandez-Munain C (2012). Tcra enhancer activation by inducible transcription factors downstream of pre-TCR signaling. J Immunol.

[38] Oh-hora M (2009). Calcium signaling in the development and function of T-lineage cells. Immunological Reviews.

[39] Brignall R (2017). Integration of Kinase and Calcium Signaling at the Level of Chromatin Underlies Inducible Gene Activation in T Cells. J Immunol.

[40] Su RC, Sridharan R, Smale ST (2005). Assembly of silent chromatin during thymocyte development. Semin Immunol.

[41] Holden NS (2008). Phorbol ester-stimulated NF-kappaB-dependent transcription: roles for isoforms of novel protein kinase C. Cell Signal.

[42] Reizis B, Leder P (2001). The Upstream Enhancer Is Necessary and Sufficient for the Expression of the Pre-T Cell Receptor α Gene in Immature T Lymphocytes. J. Exp. Med..

[43] Da Silva, T. A., Oliveira-Brito, P. K. M., Goncalves, T. E., Vendruscolo, P. E. & Roque-Barreira, M. C. ArtinM Mediates Murine T Cell Activation and Induces Cell Death in Jurkat Human Leukemic T Cells. *Int J Mol Sci* **18** (2017). 10.3390/ijms18071400 PMC5535893 28665310

[44] Germain RN (2002). T-cell development and the CD4-CD8 lineage decision. Nat Rev Immunol.

[45] Mingueneau M (2013). The transcriptional landscape of alphabeta T cell differentiation. Nat Immunol.

[46] McLean CY (2010). GREAT improves functional interpretation of cis-regulatory regions. Nat Biotechnol.

[47] Vanden Bempt M (2018). Cooperative Enhancer Activation by TLX1 and STAT5 Drives Development of NUP214-ABL1/TLX1-Positive T Cell Acute Lymphoblastic Leukemia. Cancer Cell.

[48] Burlacu B (2003). Regulation of apoptosis by Bcl-2 family proteins. J. Cell. Mol. Med..

[49] Kirkin V, Joos S, Zornig M (2004). The role of Bcl-2 family members in tumorigenesis. Biochim Biophys Acta.

[50] Dunkle A, He YW (2011). Apoptosis and autophagy in the regulation of T lymphocyte function. Immunol Res.

[51] Hata AN, Engelman JA, Faber AC (2015). The BCL2 Family: Key Mediators of the Apoptotic Response to Targeted Anticancer Therapeutics. Cancer Discov.

[52] Gratiot-Deans J, Merino R, Nunez G, Turka LA (1994). Bcl-2 expression during T-cell development: Early loss and late return occur at specific stages of commitment to differentiation and survival. Proc. Nati. Acad. Sci. USA.

[53] Tanahashi M (2001). Effect of phorbol ester and calcium ionophore on human thymocytes. Human Immunology.

[54] Feng H (2010). T-lymphoblastic lymphoma cells express high levels of BCL2, S1P1, and ICAM1, leading to a blockade of tumor cell intravasation. Cancer Cell.

[55] Coustan-Smith, E. *et al*. Clinical Relevance of BCL-2 Overexpression in Childhood Acute Lymphoblastic Leukemia. *Blood* **87** (1996). 8562940

[56] Roberts AW, Huang D (2017). Targeting BCL2 With BH3 Mimetics: Basic Science and Clinical Application of Venetoclax in Chronic Lymphocytic Leukemia and Related B Cell Malignancies. Clin Pharmacol Ther.

[57] Bornschein S (2018). Defining the molecular basis of oncogenic cooperation between TAL1 expression and Pten deletion in T-ALL using a novel pro-T-cell model system. Leukemia.

[58] Hoffman ES (1996). Productive T-cell receptor beta-chain gene rearrangement: coincident regulation of cell cycle and clonality during development *in vivo*. Genes & Development.

[59] Kingeter LM, Paul S, Maynard SK, Cartwright NG, Schaefer BC (2010). Cutting edge: TCR ligation triggers digital activation of NF-kappaB. J Immunol.

[60] Aifantis I, Gounari F, Scorrano L, Borowski C, von Boehmer H (2001). Constitutive pre-TCR signaling promotes differentiation through Ca2+ mobilization and activation of NF-κB and NFAT. Nature Immunology.

[61] Voll RE (2000). NF-κB Activation by the Pre-T Cell Receptor Serves as a Selective Survival Signal in T Lymphocyte Development. Immunity.

[62] Wallaert A, Durinck K, Taghon T, Van Vlierberghe P, Speleman F (2017). T-ALL and thymocytes: a message of noncoding RNAs. J Hematol Oncol.

[63] Orom UA, Shiekhattar R (2013). Long noncoding RNAs usher in a new era in the biology of enhancers. Cell.

[64] Liu, S. J. *et al*. CRISPRi-based genome-scale identification of functional long noncoding RNA loci in human cells. *Science* **355** (2017). 10.1126/science.aah7111 PMC5394926 27980086

[65] Guttman M (2011). lincRNAs act in the circuitry controlling pluripotency and differentiation. Nature.

[66] Ivaldi, M. S. *et al*. Fetal gamma-globin genes are regulated by the BGLT3 long non-coding RNA locus. *Blood* (2018). 10.1182/blood-2018-07-862003 PMC6213316 30150205

[67] Cho SW (2018). Promoter of lncRNA Gene PVT1 Is a Tumor-Suppressor DNA Boundary Element. Cell.

[68] Natoli G, Andrau JC (2012). Noncoding transcription at enhancers: general principles and functional models. Annu Rev Genet.

[69] Paralkar VR (2016). Unlinking an lncRNA from Its Associated cis Element. Mol Cell.

[70] Engreitz JM (2016). Local regulation of gene expression by lncRNA promoters, transcription and splicing. Nature.

[71] Sentman CL, Shutter JR, Hockenbery D, Kanagawa O, Korsmeyer SJ (1991). bcl-2 Inhibits Multiple Forms of Apoptosis but Not Negative Selection in Thymocytes. cell.

[72] Akashi K, Kondo M, von Freeden-Jeffry U, Murray R, Weissman IL (1997). Bcl-2 Rescues T Lymphopoiesis in Interleukin-7 Receptor–Deficient Mice. cell.

[73] Maraskovsky E (1997). Bcl-2 Can Rescue T Lymphocyte Development in Interleukin-7 Receptor–Deficient Mice but Not in Mutant rag-1−/−Mice. cell.

[74] Strasser A, Harris AW, Corcoran LM, Cory S (1994). Bcl-2 expression promotes B- but not T-lymphoid development in scid mice. nature.

[75] Veis DJ, Sorenson CM, Shutter JR, Korsmeyer SJ (1993). Bcl-2–deficient mice demonstrate fulminant lymphoid apoptosis, polycystic kidneys, and hypopigmented hair. cell.

[76] Mandal M (2005). The BCL2A1 gene as a pre-T cell receptor-induced regulator of thymocyte survival. J Exp Med.

[77] Murga C, Barber DF (2002). Molecular Mechanisms of Pre-T Cell Receptor-induced Survival. Journal of Biological Chemistry.

[78] Guidos CJ (1996). V(D)J recombination activates a p53-dependent DNA damage checkpoint in scid lymphocyte precursors. Genes & Development.

[79] Fotedar R (1999). Effect ofp21(waf1/cip1) transgene on radiation induced apoptosis in T cells. Oncogene.

[80] Haks MC, Krimpenfort P, van den Brakel JHN, Kruisbeek AM (1999). Pre-TCR Signaling and Inactivation of p53 Induces Crucial Cell Survival Pathways in Pre-T Cells. Immunity.

[81] Berga-Bolanos R, Alberdi M, Buxade M, Aramburu J, Lopez-Rodriguez C (2013). NFAT5 induction by the pre-T-cell receptor serves as a selective survival signal in T-lymphocyte development. Proceedings of the National Academy of Sciences.

[82] Hanahan D, Weinberg RA (2011). Hallmarks of cancer: the next generation. Cell.

[83] Strasser A (2005). The role of BH3-only proteins in the immune system. Nat Rev Immunol.

[84] Adams JM, Cory S (2007). The Bcl-2 apoptotic switch in cancer development and therapy. Oncogene.

[85] Peirs S (2014). ABT-199 mediated inhibition of BCL-2 as a novel therapeutic strategy in T-cell acute lymphoblastic leukemia. blood.

[86] Sanda T (2013). TYK2-STAT1-BCL2 pathway dependence in T-cell acute lymphoblastic leukemia. Cancer Discov.

[87] Knoechel B (2014). An epigenetic mechanism of resistance to targeted therapy in T cell acute lymphoblastic leukemia. Nat Genet.

[88] Hu G (2013). Expression and regulation of intergenic long noncoding RNAs during T cell development and differentiation. Nat Immunol.

[89] Thorvaldsdottir H, Robinson JT, Mesirov JP (2013). Integrative Genomics Viewer (IGV): high-performance genomics data visualization and exploration. Brief Bioinform.

[90] Lin S (2014). Comparison of the transcriptional landscapes between human and mouse tissues. Proceedings of the National Academy of Sciences.

[91] Trapnell C (2013). Differential analysis of gene regulation at transcript resolution with RNA-seq. Nat Biotechnol.

[92] Kryuchkova-Mostacci N, Robinson-Rechavi M (2017). A benchmark of gene expression tissue-specificity metrics. Brief Bioinform.

[93] Ito K, Murphy D (2013). Application of ggplot2 to Pharmacometric Graphics. CPT Pharmacometrics Syst Pharmacol.

[94] Reimand J, Arak T, Vilo J (2011). g:Profiler–a web server for functional interpretation of gene lists (2011 update). Nucleic Acids Res.

[95] Subramanian A (2005). Gene set enrichment analysis: a knowledge-based approach for interpreting genome-wide expression profiles. Proc Natl Acad Sci USA.

[96] Subramanian A, Kuehn H, Gould J, Tamayo P, Mesirov JP (2007). GSEA-P: a desktop application for Gene Set Enrichment Analysis. Bioinformatics.

[97] Naito Y, Hino K, Bono H, Ui-Tei K (2015). CRISPRdirect: software for designing CRISPR/Cas guide RNA with reduced off-target sites. Bioinformatics.

