# Supplementary material for: A critical regulator of Bcl2 revealed by systematic transcript discovery of lncRNAs associated with T-cell differentiation
Source: Sci Rep. 2019 Mar 18;9:4707. doi: 10.1038/s41598-019-41247-5 (PMC6423290; doi:10.1038/s41598-019-41247-5)
Supplement: Supplementary file 1 — supplementals [file 41598_2019_41247_MOESM1_ESM.pdf]

A critical regulator of Bcl2 revealed by systematic transcript discovery of lncRNAs associated with T-cell differentiation

Wiam Saadi, Yasmina Kermezli, Lan T.M. Dao, Evelyne Mathieu, David Santiago-Algarra, Iris Manosalva, Magali Torres, Mohamed Belhocine, Lydie Pradel, Beatrice Loriod, Mourad Aribi, Denis Puthier, Salvatore Spicuglia

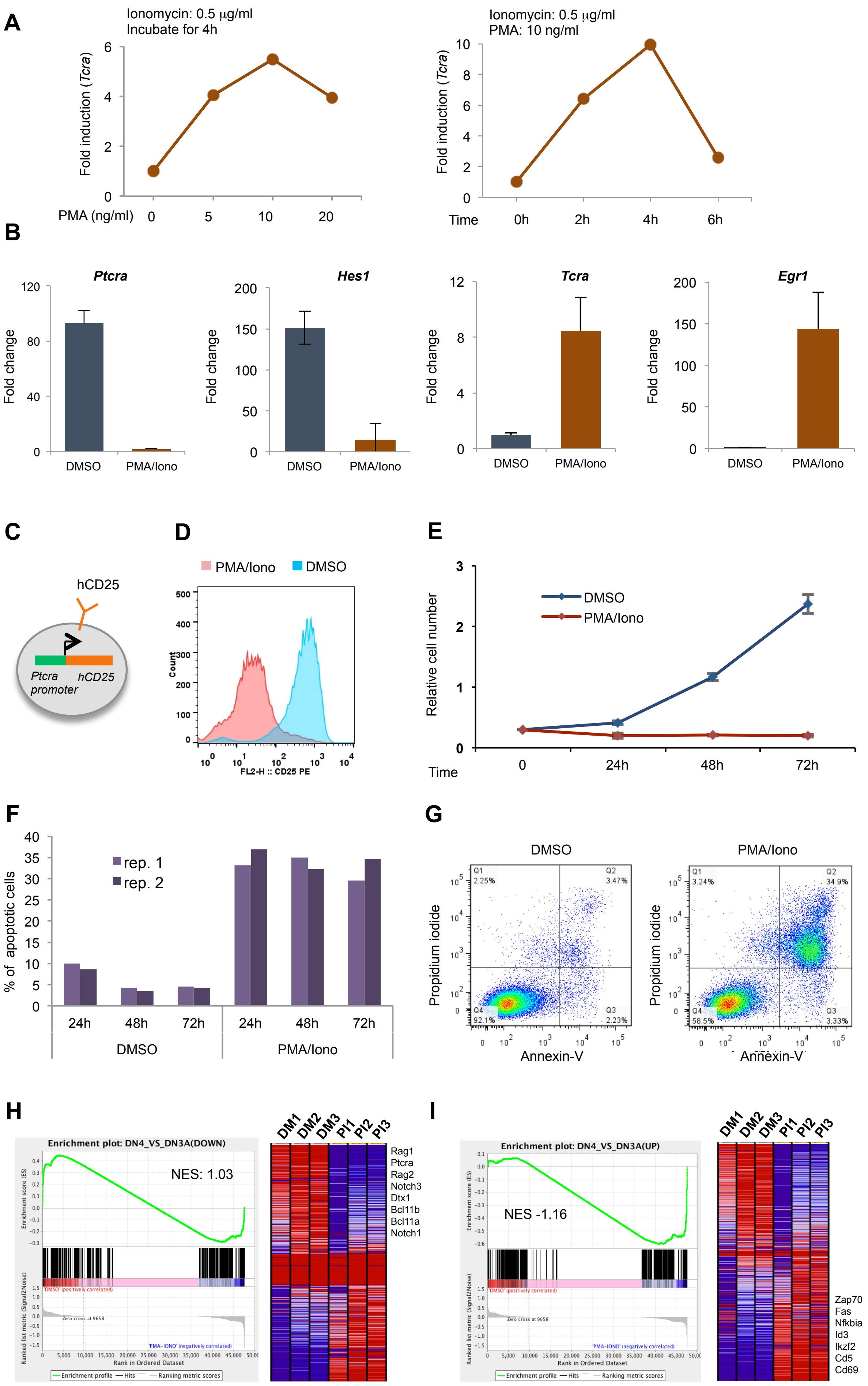

A
Ionomycin: 0.5 μg/ml
Incubate for 4h
Fold induction (Tcra)
PMA (ng/ml)
0
5
10
20
Ionomycin: 0.5 μg/ml
PMA: 10 ng/ml
Fold induction (Tcra)
Time
0h
2h
4h
6h
B
Ptcra
Hes1
Tcra
Egr1
Fold change
DMSO
PMA/Iono
C
hCD25
Ptcra promoter
hCD25
D
PMA/Iono
DMSO
Count
FL2-H :: CD25 PE
E
DMSO
PMA/Iono
Relative cell number
Time
0
24h
48h
72h
F
rep. 1
rep. 2
% of apoptotic cells
24h
48h
72h
DMSO
PMA/Iono
G
DMSO
PMA/Iono
Propidium iodide
Annexin-V
Q1 2.25%
Q2 3.47%
Q3 2.23%
Q4 92.1%
Q1 3.24%
Q2 34.9%
Q3 3.33%
Q4 58.5%
H
Enrichment plot: DN4_VS_DN3A(DOWN)
NES: 1.03
DM1
DM2
DM3
PI1
PI2
PI3
Rag1
Ptcra
Rag2
Notch3
Dtx1
Bcl11b
Bcl11a
Notch1
I
Enrichment plot: DN4_VS_DN3A(UP)
NES -1.16
DM1
DM2
DM3
PI1
PI2
PI3
Zap70
Fas
Nfkbia
Id3
Ikzf2
Cd5
Cd69

Supplementary Figure 1. A) Incubation time and dose dependent effects of the PMA/ionomycin treatment on the *Tcra* expression in P5424 cells. *Tcra* expression levels were measured by qPCR. **B)** Barplots of RT-qPCR analysis showing an induction of *Tcrα* and *Egr1*, as well as a repression of *Ptcra* and *Hes1* in P5424 cells upon PMA/ionomycin stimulation. **C)** Schematic representation of the P5424 reporter cell line expressing the hCD25 surface marker via the *Ptcra* promoter. **D)** Cytometry analysis histograms showing the reduction of the hCD25 at the cell surface in presence of PMA/ionomycin. **E)** Proliferation curve of P5424 cells treated with DMSO or PMA/ionomycin for 4h. Cells were counted at the indicated time points. **F)** Histogram showing the percentage of apoptotic P5424 cells (Annexin-V and Propidium iodide double positive cells) after PMA/ionomycin treatment in two independent experiments (rep). **G)** Representative cytometry analysis of P5424 cells stained with propidium iodide and Annexin-V after 48 hours of PMA/ionomycin treatment. **H-I)** Gene set enrichment analysis (GSEA) of repressed (**H**) and induced (**I**) genes between DN4 and DN3a thymocytes in DMSO versus PMA/ionomycin treated P5424 cells. Heat maps showing the relative gene expression of the top 50 genes of each gene set (red = high, blue = low) in DMSO (DM, 3 replicates) and PMA/ionomycin (P**I**, 3 replicates)-treated P5424 cells.

A

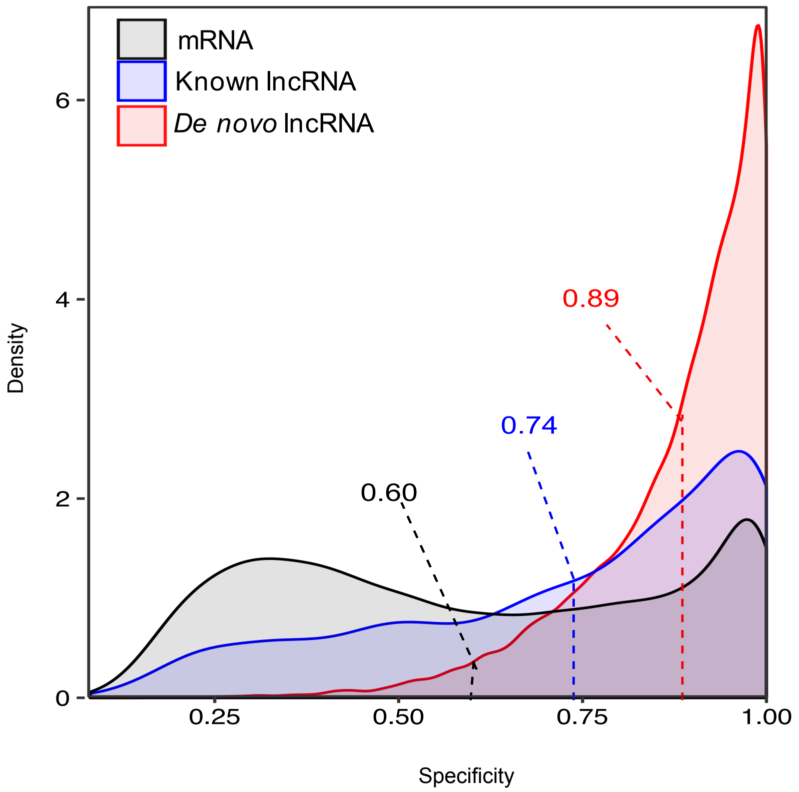

B

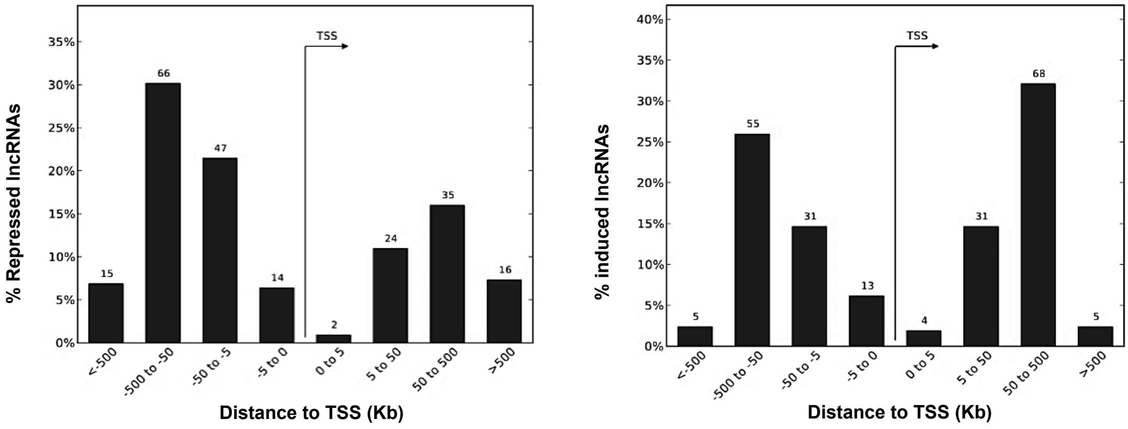

Supplementary Figure 2. A) Density plots showing the tissue-specificity scores distribution for each transcript class. Vertical lines indicate the mean tissue-specificity score of the corresponding class. **B)** Distance of the induced and the repressed lncRNAs relative to the TSS of associated coding genes using the GREAT tool.

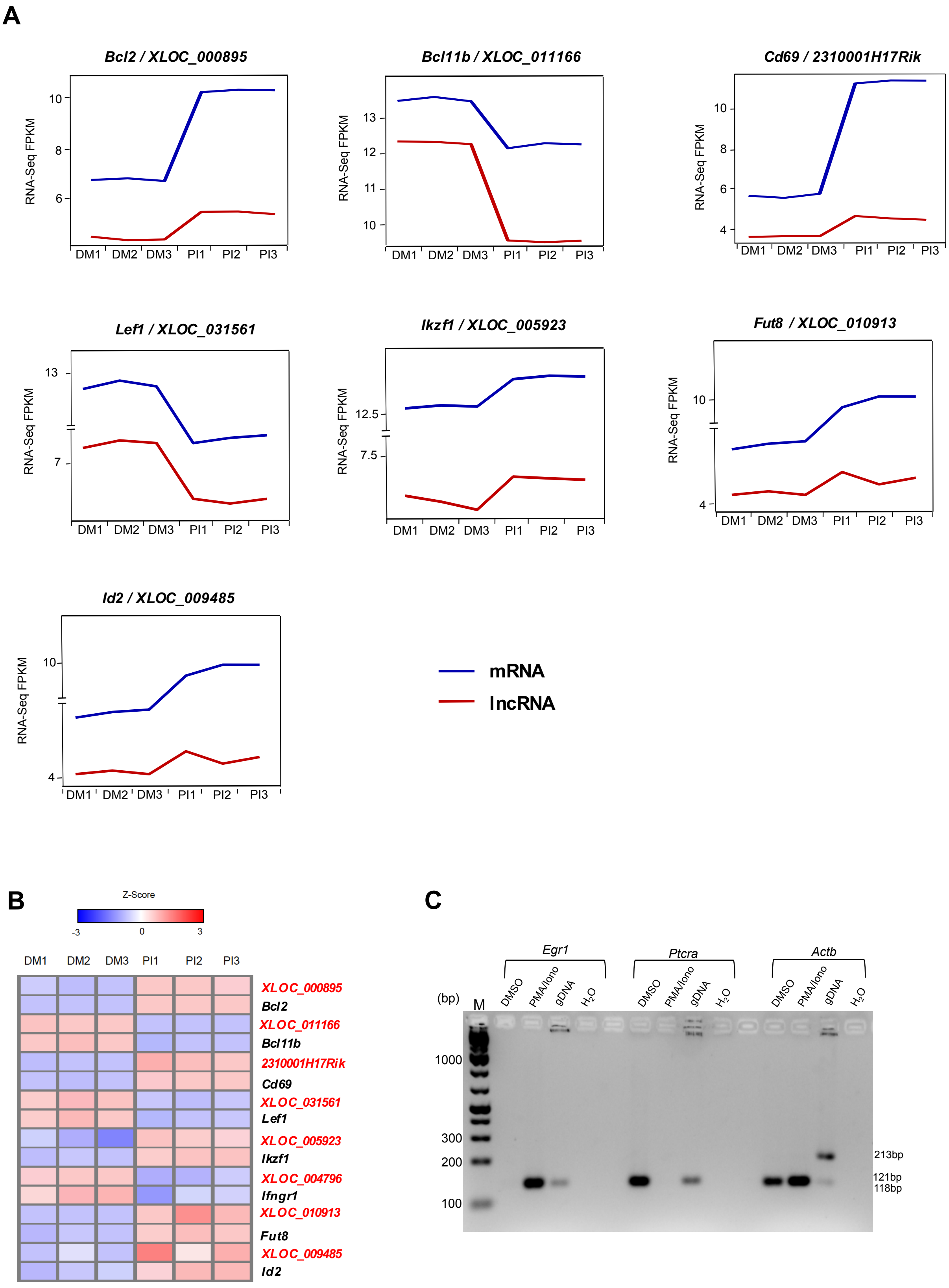

Supplementary Figure 3. A) Line plots showing the expression levels of co-regulated pairs across samples. **B)** Heat maps of co-regulated pairs showing their co-repression or their co-induction across two conditions (DMSO (DM), PMA/ionomycin (PI)). **C)** Conventional PCR amplification of *Egr1* and *Ptcra* genes. *Actb* is an internal control gene and the genomic DNA (gDNA) is an experimental control.

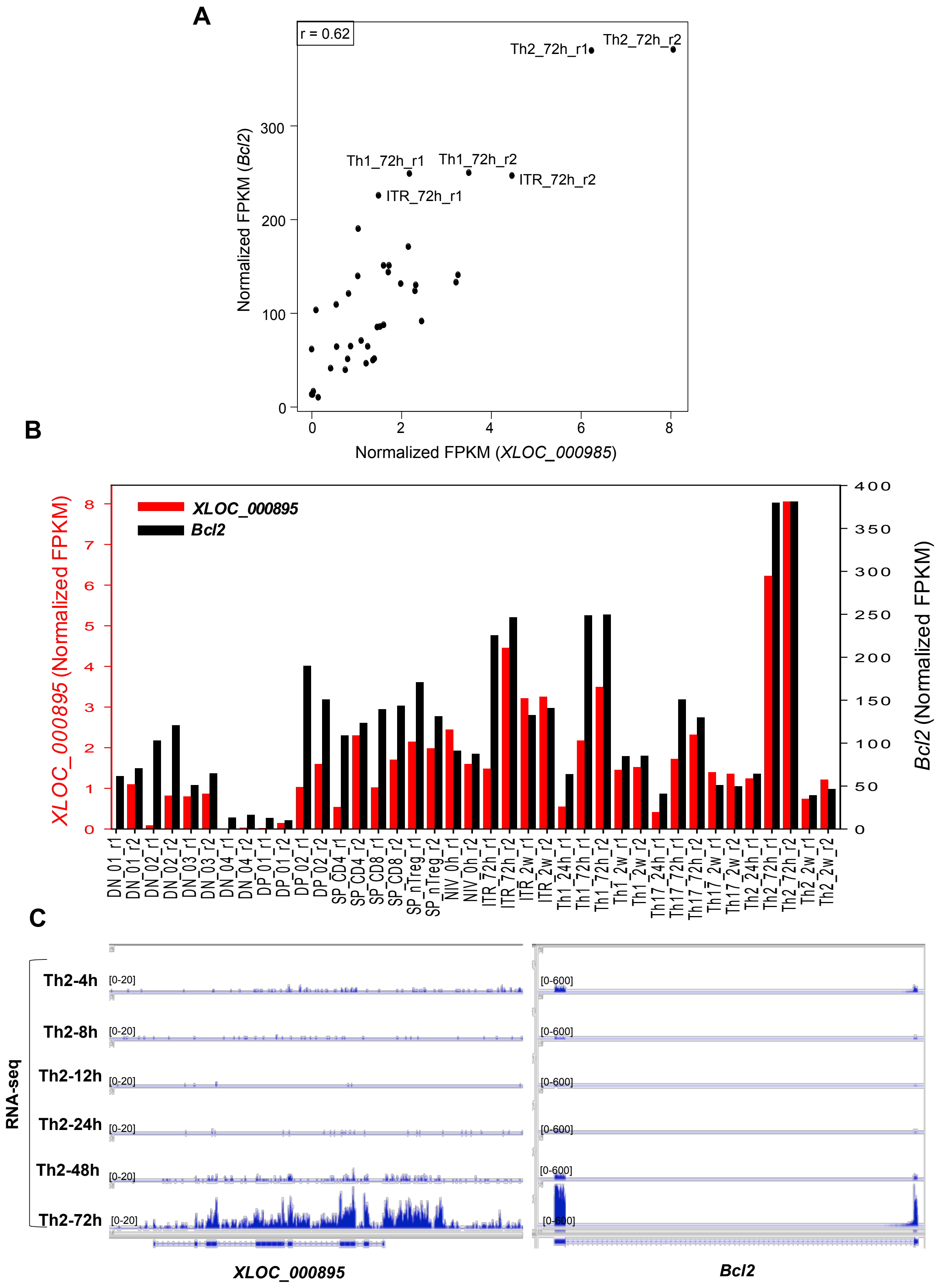

Supplementary Figure 4. A) Scatter plot showing the correlation between *Xloc_000895* and *Bcl2* expressions in mouse T-cell populations using RNA-seq data from Hu et al. 2013. **B)** Bar plots showing the expression levels of *Bcl2* and *XLOC_000895* in each mouse T-cell differentiation stage. **C)** IGV genome browser screenshots displaying tracks for RNA-seq data at the *XLOC_000895* and *Bcl2* loci during the in vitro Th2 differentiation.

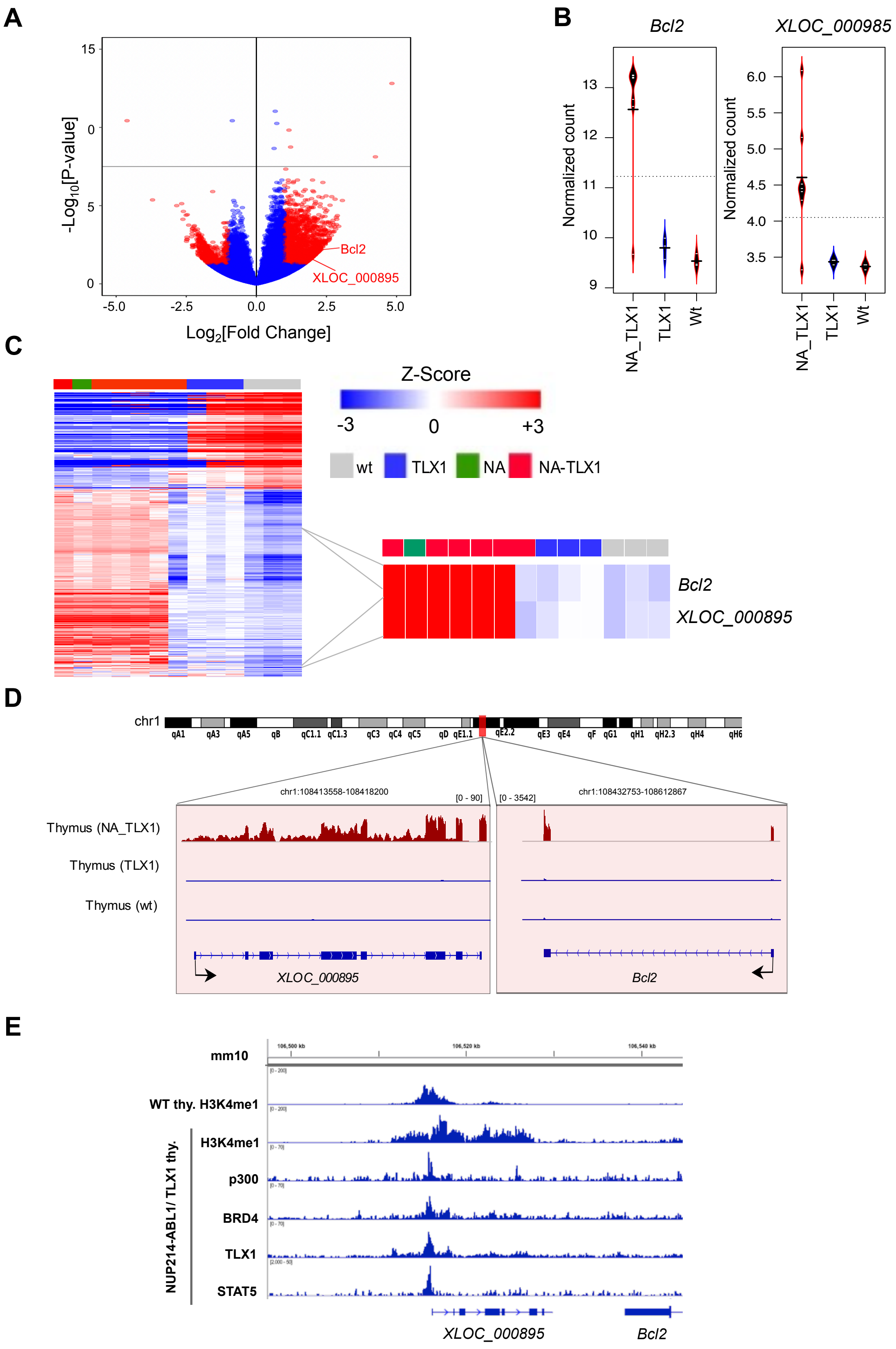

Supplementary Figure 5. A) Volcano plot depicting genes differentially expressed genes between leukemia and wildtype samples (red dots). The *Bcl2* and *XLOC_000895* are highlighted. **B)** Bean plots illustrating the expression distribution of *Bcl2* (left panel) and *XLOC_000895* (right panel) in different transgenic mouse models (NA = NUP214/ABL1). **C)** Heat maps of differentially expressed genes between wildtype and leukemic models. **D-E)** Genome browser screenshots displaying the RNA-seq signal in leukemic and normal thymocytes on the *XLOC_000895* and *Bcl2* loci (the scales are indicated on the top of each panel). (**D**) and the genomic coverage of epigenetic marks and transcription factors near the *XLOC_000895* promoter (**E**).

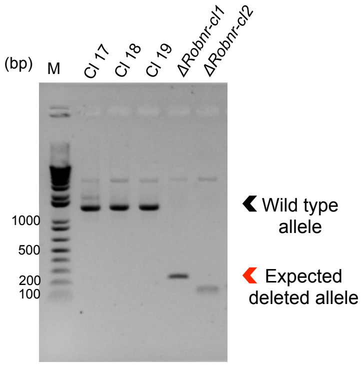

Supplementary Figure 6. Assessment of successful *Robnr* deletion by classic PCR validation in corresponding P5424 clones.

Supplementary Table 1: Oligonucleotides sequences used in this study

Primer sequences used for classic PCR:

Primer name	Primer sequence	Annealing temperature
Egr-1	R:TTCTTTATGCCAACTTGATGGTCTAG<br>F: CTTCACCTCTATCCAAAGGACTTGA	60°
Ptα	R: TGCCATTGCCAGCTGAGA<br>F:CTGGCTCCACCCATCACACT	60°
Act-b	R:CATGTCGTCCCAGTTGGTAA<br>F:GCTCTTTTCCAGCCTTCCTT	60°
XLOC_011166	R:AGGTGACAGGGCCTTTCTTG<br>F:GAGACCAGGAAGCCACTGTC	63°
XLOC_031561	R:CGGCTGGTGTTTGCAGTTAG<br>F: CTGCTCAGTCACCCCTTTGT	62°
XLOC_004796	R:GATCCAGTTCTGTGTGTGAGTCT<br>F: ACAGGGCTCCTCAGGAAAGA	62°
XLOC_000895	R:TGCTGCTCTGGGATCTCTCA<br>F:CCGGACATGTGTTCAGTCTCA	64°
NR_040266	R:TGGTGGCTGACATTCATTCCT<br>F: TCCTGTGGCAGCTGAATTCC	64°
XLOC_005923	R: AGACACTGAGATGGGAAGGGA<br>F: CAAAGGGAGCTGGGGATGAG	62°
XLOC_010913	R: TGCTAGGATTCAGGCTTGGTG<br>F:AGCTGTGTAGGCAAGTGAGC	60°
XLOC_009485	R:AAGGGTGGGGAAAGGATGGA<br>F:CACCCTGACACTCTCGAAGC	60°

Primer sequences used for RT-qPCR

Primer name	Primer sequence	Annealing temperature
Egr-1	R:TTCTTTATGCCAACTTGATGGTCTAG<br>F: CTTCACCTCTATCCAAAGGACTTGA	60°
Ptα	R: TGCCATTGCCAGCTGAGA<br>F:CTGGCTCCACCCATCACACT	60°
Act-b	R:CATGTCGTCCCAGTTGGTAA<br>F:GCTCTTTTCCAGCCTTCCTT	60°
Tcrα	R:GAGGATTCGGAGTCCCATAAC<br>F:AAAGAGACCAACGCCACCTAC	60°
XLOC_011166	F:TGGTTATCAGGACAGTGAGCAC<br>R:AGGTGACAGGGCCTTTCTTG	60°
Bcl11b	R:TGGGAAGAGGAGGCAGCTAT<br>F:AAAGGCATCTGTCCCAAGCA	60°
XLOC_031561	R:CGGGTACAGGGACCCTTCTA<br>F:CTGCTCTCACACTGGCTGAA	60°
Lef1	R: GGCTTGTCTGACCACCTAATG<br>F:TCTTCGCCGAGATCAGTCAT	60°
XLOC_004796	R:CGCTCCTGTAATTTGTCCTCCT<br>F: ACAGGGCTCCTCAGGAAAGA	60°
Ifngr1	R: TCCTTCTTCCTCCTGATCTCCA<br>F:GGGCCAGAGTTAAAGCTAAGGT	60°
XLOC_000895	R:TGCTGCTCTGGGATCTCTCA<br>F:CCGGACATGTGTTCAGTCTCA	64°
Bcl2	R: GCTGGGGCCATATAGTTCCA<br>F:GAGAGCGTCAACAGGGAGAT	60°
NR_040266	R: ACCCTCCAAGCTTTGTATGCA<br>F:TCCTGTGGCAGCTGAATTCC	60°
Cd69	R: CCAGAATATCGCTTCAGAAACGT<br>F:TCTCCACCACAACCAAGAGT	60°
XLOC_005923	F:TCCTACTTTTCTGTACCCTCCCA<br>R: AGACACTGAGATGGGAAGGGA	60°
Ikzf1	R: GCTCATCCCCTTCATCTGGA<br>F:TGGATGTCGATGAGGGTCAA	60°
Xloc _010913	R:TCCAGGTGATGAATGTAGCTCC<br>F:AGCTGTGTAGGCAAGTGAGC	60°
Fut8	R:GAAGGCTGCTTCTGTTCCCA<br>F:CCACAACCTTGGCTGGAAAAG	60°
XLOC_009485	R: TTCTACAGAGGAGGTGCGGT<br>F: AGAAGGCAAAGCTAGCTCGG	60°

Id2	R: GCCACAGAGTACTTTGCTATCA<br>F: CACCCTGAACACGGACATCA	60°

Primer sequences used for ChIP-qPCR

Name	Sequence (5'-3')	Annealing temperature
Bcl2(R1)	R: ACCTACCCAGCCTCCGTTAT<br>F:GAGAGCGTCAACAGGGAGAT	60°
Bcl2(R2)	R:GAATCGGGAGTTGGGGTCTG<br>F: TGCGGTGCTCTTGAGATCTC	
Bcl2(R3)	R:TCAGCTAACCATGTGAACCCC<br>F: TCCTGGAGACTGTGTTGGGA	

Guide RNA sequences

Name	Sequence (5'-3')	Coordinates	Expected deleted region (bp)
Bcl2_G1	CCAGGGATTTAGGTCACATATTG	chr1:108411996-108412018	1129
Bcl2_G2	AAGGAATTTAACACTTGAAGTGG	chr1:108413103-108413125	

Primer sequences for detection of genome editing

Name	Sequence (5'-3')	Cordinate	Expected band size (bp)	
			With editing	Without editing
Bcl2_F	ACACACAGGTCCAAGTCAAGG	chr1:108411879-108411899	217	1346
Bcl2_R	GCCACCTCTTTAGCCCAGAA	chr1:108413205-108413224		
